# A review of low-, medium-, and high-temperature thermoelectric materials for thermoelectric generators

**DOI:** 10.1016/j.isci.2026.117418

**Published:** 2026-09-07

**Authors:** Zhaojun Liu, Jiarun Ge, Junyu Jin, Chen Wu, Kai Wang, Qisheng Huang, Qinghua Wang, Zhongkai Zhang, Bian Tian, Zhuangde Jiang

**Affiliations:** 1 State Key Laboratory for Mechanical Manufacturing Systems Engineering, Xi’an Jiaotong University, Xi’an 710049, China

**Keywords:** temperature, thermoelectric materials, thermoelectric generators, energy recovery

## Abstract

Thermoelectric generators convert heat directly into electricity and require different material and engineering strategies across operating temperatures. This review organizes thermoelectric materials and representative generators into low-, medium-, and high-temperature regimes and evaluates their performance using material-, device-, module-, and system-level evidence. Low-temperature systems are dominated by limited temperature differences, heat-source coupling, contact resistance, and mechanical compliance. Medium-temperature technologies depend on high average zT, p-n compatibility, diffusion barriers, and segmented architectures. High-temperature systems increasingly require oxidation resistance, suppression of elemental volatilization, stable low-resistance interfaces, thermomechanical compatibility, reliable joining and packaging, and scalable manufacturing. Half-Heusler alloys, skutterudites, SiGe, Cu_2_X compounds, Zintl-related materials, and thermoelectric oxides are compared in this framework. Progress toward practical deployment will depend on translating material performance into durable modules validated under realistic thermal, mechanical, and environmental conditions.

## Graphical abstract

**Figure:**
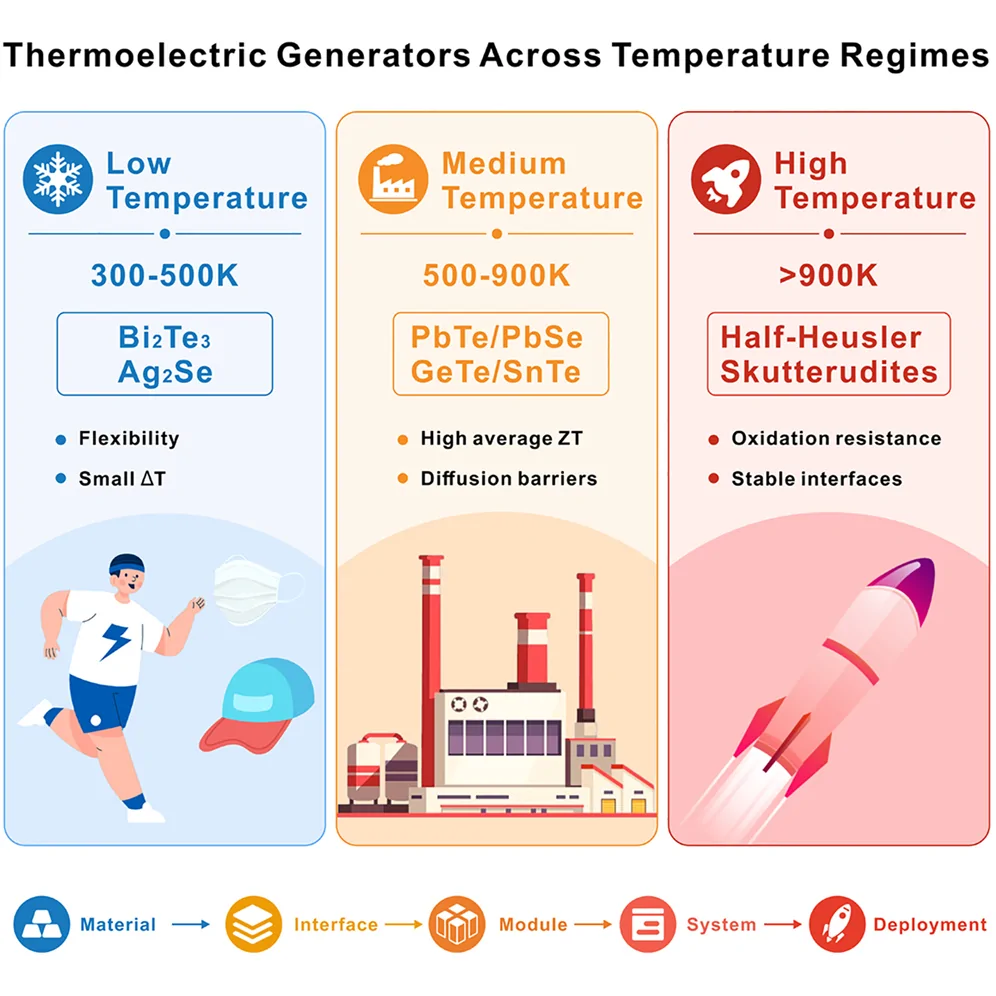

## Teaser

Electrical engineering; Materials science

## Introduction

Energy is fundamental to modern industry and daily life. Currently, the majority of energy production relies on fossil fuels.^1^^,^^2^^,^^3^^,^^4^^,^^5^ Conventionally, fuel is first burned in heat engines to generate mechanical energy, which is then converted into electrical energy via electric generators and stored for later use.^6^^,^^7^ However, this traditional pathway increasingly faces challenges in meeting the rising energy demand while addressing environmental constraints. Consequently, attention has shifted toward the development and efficient utilization of renewable energy sources.^8^^,^^9^^,^^10^^,^^11^^,^^12^^,^^13^^,^^14^^,^^15^^,^^16^^,^^17^^,^^18^ Renewable energy can be broadly categorized into two types. One is sourced directly from natural resources, including solar, wind, hydro, and photovoltaic power.^19^^,^^20^^,^^21^^,^^22^^,^^23^ The other is derived from recovering energy losses produced by human activities,^24^ representing an important approach for sustainable energy utilization.

Energy losses are ubiquitous and unavoidable, occurring throughout daily life and often accounting for the largest portion of total energy consumption.^25^^,^^26^^,^^27^^,^^28^^,^^29^^,^^30^^,^^31^^,^^32^^,^^33^ Consequently, the effective collection and reutilization of these losses represents a critical strategy for reducing thermal waste and mitigating environmental impacts.^34^ In this context, research on thermoelectric materials and devices has garnered increasing attention, providing a promising approach for efficient energy harvesting.^35^^,^^36^^,^^37^^,^^38^^,^^39^^,^^40^^,^^41^^,^^42^ Thermoelectric generators (TEGs) convert an applied temperature difference directly into electrical energy via the thermoelectric effect, a phenomenon first reported by Thomas Johann Seebeck in 1821. Seebeck showed that a temperature gradient between two dissimilar materials produces a thermoelectric potential, a principle now referred to as the Seebeck effect.^43^ Over the past decades, various TEG architectures have been developed to satisfy diverse power requirements across different applications.^44^^,^^45^^,^^46^^,^^47^^,^^48^^,^^49^ Thermoelectric materials can be broadly categorized into inorganic, organic, and organic-inorganic hybrid types, with inorganic materials generally exhibiting superior overall performance and higher thermoelectric figures of merit.^50^^,^^51^^,^^52^^,^^53^ Among them, bismuth telluride (Bi_2_Te_3_), antimony telluride (Sb_2_Te_3_), and various doped derivatives have demonstrated outstanding thermoelectric performance, with Bi_2_Te_3_ exhibiting particularly high efficiency at room temperature.^54^^,^^55^^,^^56^^,^^57^^,^^58^^,^^59^^,^^60^ The performance of a TEG is governed by a hierarchy of material-, interface-, module-, and system-level factors. Material properties determine the intrinsic thermoelectric potential, whereas contact resistance, p-n leg compatibility, module geometry, heat transfer, and packaging determine how effectively this potential is converted into usable electrical power. The present review primarily focuses on thermoelectric materials and their temperature-dependent performance. Representative generators and modules are included to illustrate the translation of material advances into device-level performance rather than to provide an exhaustive review of complete thermoelectric systems.

TEGs were initially developed mainly in bulk configurations. Although bulk devices provided effective thermoelectric conversion, their rigid structures limited their use in applications requiring miniaturization, mechanical compliance, and system integration.^61^^,^^62^^,^^63^ With the development of microelectromechanical systems in the 1990s, thin-film TEGs fabricated on rigid substrates enabled more compact device architectures. Since the early 2000s, increasing attention has been directed toward flexible thin-film TEGs that combine thermoelectric materials with flexible electronics and microfabrication technologies. These devices can conform to curved or irregular heat-source surfaces and are, therefore, attractive for wearable electronics, distributed sensing, and compact waste-heat recovery.^64^^,^^65^^,^^66^^,^^67^^,^^68^^,^^69^^,^^70^^,^^71^

In addition to device configuration, operating temperature is a fundamental criterion for thermoelectric material selection and generator design. In this review, thermoelectric materials and representative generators are discussed in low-, medium-, and high-temperature regimes according to their principal application conditions. The temperature boundaries are not absolute because the operating ranges of several material families overlap. Nevertheless, this classification provides a useful framework for identifying the dominant material-, interface-, and system-level constraints that emerge as the operating temperature increases.

The central perspective of this review is that increasing operating temperature changes not only the appropriate thermoelectric material system but also the dominant loss mechanisms and the hierarchy of design priorities. At low temperatures, practical output is mainly limited by the small available temperature difference, thermal coupling, electrical contact resistance, and mechanical compliance. At medium temperatures, the emphasis shifts toward a high average ZT, broad-temperature-range stability, p-n material compatibility, and interfacial diffusion. At high temperatures, oxidation, elemental volatilization, interfacial reactions, thermomechanical stress, joining, and packaging increasingly determine module efficiency and operational lifetime. The following sections, therefore, evaluate thermoelectric development according to this temperature-dependent transition from material-dominated limitations to coupled material-interface-module-system constraints.

Recent studies indicate that high-temperature TEGs represent a critical frontier for future research. Although both the materials and structural designs of low- and medium-temperature TEGs are well established, high-temperature applications remain challenging due to extreme working conditions.^72^^,^^73^ TEGs are commonly classified into low-, medium-, and high-temperature ranges based on their operating temperature, each corresponding to distinct application scenarios.

Low-temperature thermoelectric systems are primarily used in wearable electronics, Internet-of-Things sensors, and low-grade heat recovery near room temperature. Their performance is strongly influenced by the limited available temperature difference, thermal contact with the heat source, device flexibility, and electrical contact resistance. Bi_2_Te_3_-based compounds, Ag_2_Se, and conducting polymers are representative material systems in this regime.^74^^,^^75^^,^^76^^,^^77^^,^^78^^,^^79^^,^^80^^,^^81^^,^^82^^,^^83^^,^^84^^,^^85^^,^^86^^,^^87^^,^^88^^,^^89^^,^^90^^,^^91^^,^^92^^,^^93^^,^^94^^,^^95^^,^^96^^,^^97^^,^^98^ Medium-temperature systems are more relevant to automotive exhaust, boilers, and industrial waste-heat sources. Their development requires a high average ZT over a broad temperature range, chemically and mechanically stable interfaces, and scalable module fabrication. Representative materials include PbTe-, PbSe-, GeTe-, SnTe-, and Mg_3_Sb_2_-based compounds and skutterudites.^99^^,^^100^^,^^101^^,^^102^^,^^103^^,^^104^^,^^105^^,^^106^^,^^107^^,^^108^^,^^109^

High-temperature TEGs, designed for industrial waste-heat recovery, remain less developed.^110^^,^^111^ High-temperature TEGs are intended for industrial furnaces, concentrated-solar systems, gas turbines, and aerospace environments. Compared with low- and medium-temperature systems, their development is constrained not only by thermoelectric transport properties but also by intrinsic carrier excitation, bipolar transport, elemental volatilization, oxidation, interfacial diffusion, thermal-expansion mismatch, and high-temperature packaging. Candidate material systems include half-Heusler (HH) alloys, skutterudites, SiGe alloys, Cu_2_X compounds, Zintl phases, and thermoelectric oxides.^112^^,^^113^^,^^114^^,^^115^^,^^116^^,^^117^^,^^118^^,^^119^^,^^120^^,^^121^^,^^122^^,^^123^^,^^124^^,^^125^^,^^126^^,^^127^ Consequently, high-temperature generator development requires coordinated optimization at the material, interface, module, and thermal management levels.

**Figure 1:**
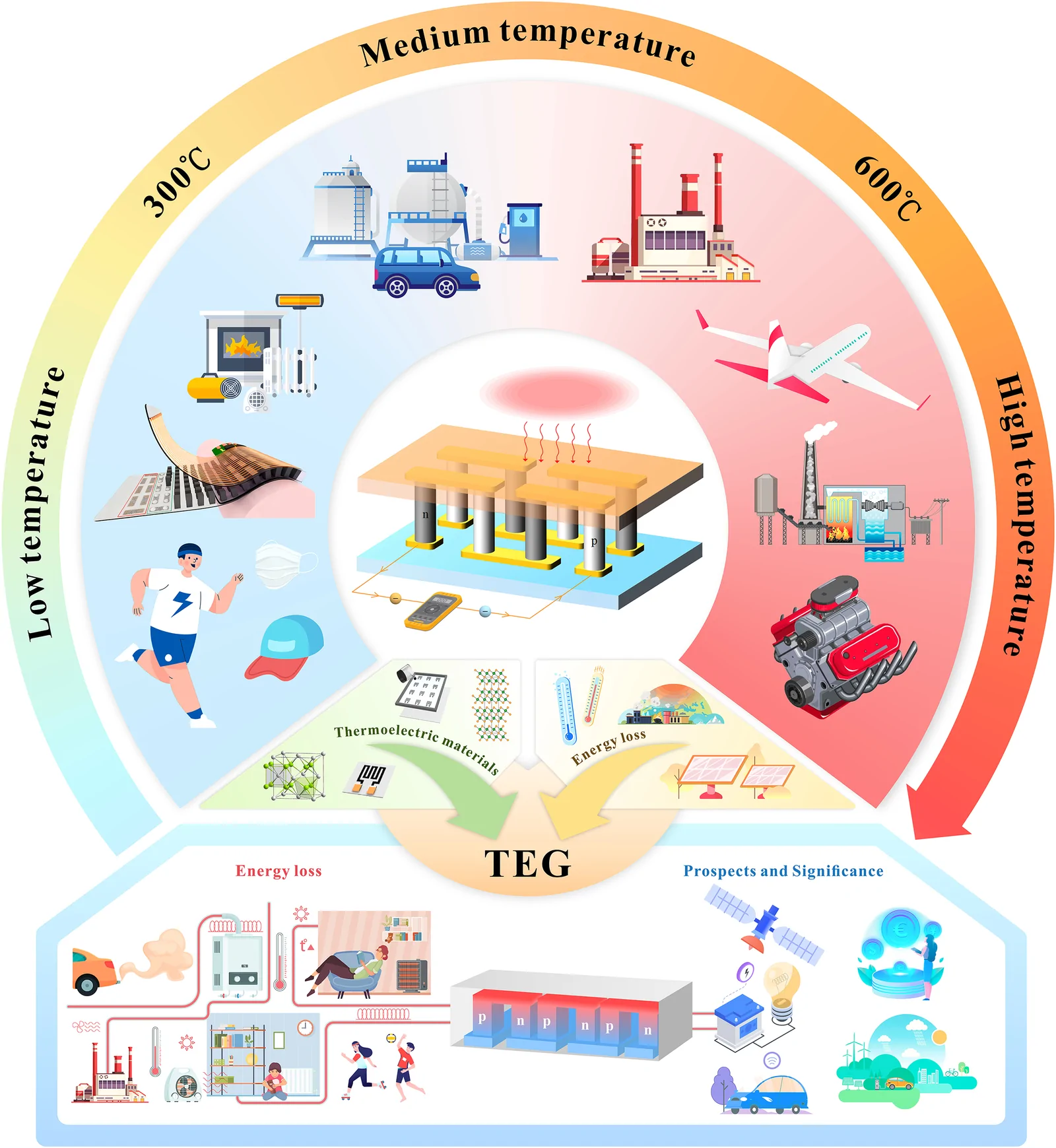
Temperature regimes, representative thermoelectric materials, device configurations, and application scenarios of thermoelectric generators

Figure 1 summarizes the representative material systems, device configurations, and application scenarios across the low-, medium-, and high-temperature regimes considered in this review. In this review, we first introduce the fundamental thermoelectric parameters that govern material and generator performance. Thermoelectric materials are then discussed according to low-, medium-, and high-temperature operating regimes, with emphasis on representative compositions, transport-property regulation, fabrication methods, and performance over the relevant temperature ranges. Device and module demonstrations are selectively included to assess whether improvements in material ZT, power factor, or thermal stability have been translated into enhanced output power, conversion efficiency, or operational reliability. Particular attention is given to high-temperature materials because their practical use is strongly affected by intrinsic excitation, bipolar transport, oxidation, elemental volatilization, interfacial diffusion, thermal-expansion mismatch, and packaging. Finally, the transferability of material and interface strategies across temperature regimes is examined and the research priorities for reliable and scalable high-temperature TEGs are discussed.

## Principles of thermoelectric theory

Thermoelectric materials directly couple heat and charge transport through the Seebeck, Peltier, and Thomson effects. When a temperature gradient is applied to a thermoelectric material, charge carriers diffuse from the hot region toward the cold region, producing an electrical potential. The Seebeck coefficient S describes the voltage generated per unit temperature difference. To construct a TEG, p-type and n-type thermoelectric legs are connected electrically in series and thermally in parallel through metallic electrodes. Material-level transport parameters determine the intrinsic conversion potential, whereas leg geometry, electrical contact resistance (ECR) and thermal contact resistance (TCR), interconnects, and heat-transfer conditions influence the measured generator performance.^128^^,^^129^ This redistribution generates a potential difference across the device, known as the thermoelectric voltage (V). The Seebeck coefficient (S) quantifies the voltage generated per unit temperature difference, as expressed by:

(Equation 1) V=S×ΔT

The maximum conversion efficiency of a practical TEG differs from that predicted for an isolated homogeneous thermoelectric leg because additional energy exchange occurs at the interfaces between the thermoelectric materials and the metallic electrodes. When an electric current crosses such an interface, the difference between the Seebeck coefficients of the thermoelectric material and the electrode produces interfacial Peltier heat. At the cold-side interface, the Peltier heat can be expressed as q_c_ = ΔST_c_J, where ΔS = S_TE_ − S_metal_ is the difference in Seebeck coefficients between the thermoelectric material and the metallic electrode, *T*_*c*_ is the cold-side absolute temperature, and *J* is the electric current density. A portion of this interfacial Peltier heat flows back into the thermoelectric leg, whereas the remaining portion is released directly to the cold-side reservoir. This additional heat-transfer contribution, which bypasses useful thermoelectric conversion, has been termed the “heat-leap” effect.^130^

To describe the partition of the cold-side interfacial Peltier heat, a dimensionless heat-leap coefficient λ is introduced. The coefficient λ represents the fraction of q_c_ that is directly released at the cold side and consequently requires an additional heat input of λq_c_ from the hot side to maintain the prescribed temperature difference. According to energy conservation, the total heat input required at the hot side is, therefore, E_h_ = P + Q_c_ + λq_c_, where P is the electrical output power and Q_c_ is the heat transferred through the thermoelectric leg to the cold side. The conversion efficiency should consequently be evaluated as η = P/E_h_, by maximizing this efficiency with respect to the electric current; the maximum conversion efficiency considering the heat-leap effect can be expressed as:

(Equation 2) $$\eta_{\max}=\left(\frac{T_{h}{-T}_{c}}{T_{h}+\lambda T_{c}}\right)\times \frac{\sqrt{1+ZT+\lambda ZT_{c}}-1}{\sqrt{1+2ZT+\lambda ZT_{c}}+\frac{T_{c}\left(\lambda +1\right)}{T_{h}+\lambda T_{c}}}$$

where T_h_ and T_c_ are the absolute temperatures at the hot and cold sides, respectively, and *Z* = *σS*^2^/*κ* is the dimensional thermoelectric figure of merit. The coefficient λ characterizes the partition of the interfacial Peltier heat and is not an intrinsic thermoelectric material property. Song et al. suggested λ ≈ 0.5, indicating that approximately half of the cold-side interfacial Peltier heat was released directly at the cold end. Their analysis further showed that the heat-leap effect could reduce the conversion efficiency of representative TEG units by approximately 10% compared with the efficiency predicted using the conventional material-level expression. The value of λ should, nevertheless, be experimentally determined or calibrated for different device configurations and interfacial conditions rather than treated as a universal constant.

Equation 2 is derived under one-dimensional steady-state conditions and assumes temperature-independent thermoelectric transport properties. It accounts for the additional heat input associated with interfacial Peltier heat partitioning but does not explicitly include electrical contact resistance. Contact resistance reduces the output power and should, therefore, be considered separately when predicting the measured efficiency of a complete thermoelectric module.

Most metals and semiconductors can be employed for TEG fabrication to achieve enhanced performance, including high output voltage, low internal resistance, and high power output. The thermoelectric figure of merit (ZT) directly influences device efficiency,^131^ with higher ZT values corresponding to more efficient thermoelectric behavior. For a thermoelectric element, ZT is defined as:

(Equation 3) $$ZT=\frac{\sigma \times S^{2}}{\kappa }\times T$$

where σ is the electrical conductivity (S cm^−1^), κ is the thermal conductivity (W m^−1^ K^−1^), and σ × S^2^ represents the power factor. Thermal conductivity κ is closely related to lattice vibrations and charge carrier transport. Specifically, κ_l_ denotes the lattice thermal conductivity, while κ_e_ represents the electronic thermal conductivity.^132^^,^^133^^,^^134^^,^^135^ The relationship can be expressed as:

(Equation 4) $$\kappa =\kappa_{l}+\kappa_{e}$$

Electrical conductivity is given by:

(Equation 5) σ=neμ

where e is the carrier charge, μ is the carrier mobility, and n is the carrier concentration, representing the number of holes in the valence band or electrons in the conduction band. Because S and σ are strongly coupled, optimizing n is critical for achieving high σ × S^2^ and high ZT. Since κ_l_ is relatively insensitive to carrier concentration, doping is typically employed to reduce lattice thermal conductivity. Rational lattice structures and appropriate doping levels, including elemental substitution and alloying, are, therefore, key strategies to enhance thermoelectric performance. Design considerations must also account for the operational temperature range and fabrication methods to maximize performance. In general, efficient thermoelectric materials combine high electrical conductivity with low thermal conductivity to achieve high power output.^136^^,^^137^^,^^138^^,^^139^^,^^140^^,^^141^^,^^142^^,^^143^ To increase the output power of TEGs, multiple thermoelectric elements are typically connected electrically in series and thermally in parallel.

A complete TEG generally consists of multiple thermoelectric modules, each comprising numerous thermocouples. The total output voltage can be expressed as:

(Equation 6) $$V_{out}=N\times \left(S_{p}-S_{n}\right)\times \Delta T$$

where N is the number of thermocouples; S_p_ and S_n_ are the Seebeck coefficients of the p-type and n-type electrodes, respectively; and ΔT is the temperature difference across the device.

The output current and power are given by^144^^,^^145^^,^^146^:

(Equation 7) $$I=\frac{N\times \left(S_{p}-S_{n}\right)\times \Delta T}{R_{1}+R_{i}}$$

(Equation 8) $$P=I^{2}\times R_{1}$$

(Equation 9) $$P_{\max}=\frac{N^{2}\times {\left(S_{p}-S_{n}\right)}^{2}\times {\Delta T}^{2}}{4R_{i}}$$

Here, R_i_ is the internal resistance of the TEG, and R_1_ is the load resistance. While increasing the number of thermocouples raises the output voltage, it also increases the total internal resistance, which can reduce output power. Maximum power is achieved when the total internal resistance matches the load resistance; therefore, the optimal number of thermocouples must be carefully chosen to balance voltage and power output.

## Thermoelectric materials and representative generators across different temperature ranges

Throughout this section, material advances and generator demonstrations are distinguished according to the level of available evidence. Material-level performance is assessed using the Seebeck coefficient, electrical conductivity, thermal conductivity, power factor, and zT. Device-level evidence includes single-leg or unicouple output voltage, output power, power density, and contact resistance. Module- and system-level evidence further considers conversion efficiency, compatibility between p-type and n-type legs, thermal management, thermal-cycling stability, environmental durability, and auxiliary energy consumption. Accordingly, a high peak zT is treated as evidence of material potential rather than, by itself, proof of a mature TEG technology.

### Low-temperature thermoelectric materials and generators

Since 2001, extensive research efforts have been devoted to TEGs operating in the low-temperature regime, particularly for wearable applications. Both rigid and flexible TEGs have been widely investigated. Wearable TEGs are well suited for harvesting energy from human body heat, and the temperature requirements for such applications are relatively mild. Consequently, a broad range of thermoelectric materials are applicable in the low-temperature regime.^147^^,^^148^^,^^149^^,^^150^ Its core application scenarios include wearable electronics for human body temperature waste-heat recovery, Internet-of-Things passive sensing, cold chain low-temperature difference power generation, and waste-heat recovery for electronic devices. The core demand boundary of this temperature range is high power density, high flexibility, and high integration under a small temperature difference near room temperature, and the core constraint is the extremely low thermoelectric voltage caused by the small temperature difference, which puts forward the ultimate requirements for the power factor of the material, the interface contact resistance of the device, and the control of thermal loss.

Recent low-temperature thermoelectric studies can be broadly classified into material-level transport optimization, mechanically compliant device design, and improvement of heat-source coupling. Ag_2_Se- and Bi_2_Te_3_-based inorganic materials are particularly important because they combine comparatively high thermoelectric performance near room temperature with compatibility with thin-film and flexible-device fabrication.

**Figure 2:**
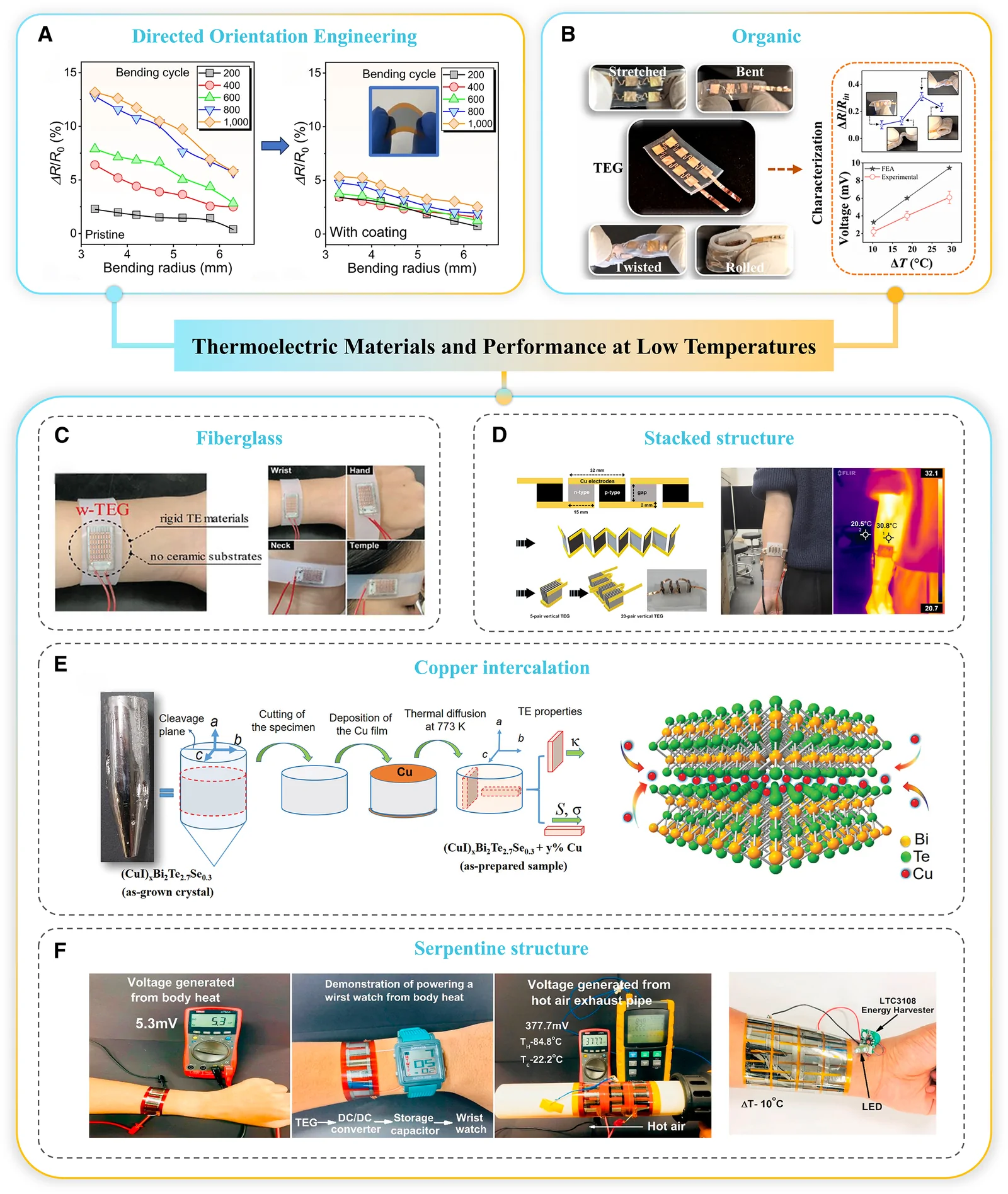
Low-temperature thermoelectric generators (A) Flexibility of Te-doped Ag_2_Se thin films and their device performance.^151^ Copyright 2024, Springer Nature. (B) Assembled TEG device. Demonstration of mechanical reliability under various deformations.^152^ Copyright 2025, Elsevier. The V_oc_ data represent averages of four repeated measurements; the source publication did not specify the statistical definition of the error bars. (C) Wearable TEGs capable of being used in multiple parts of the body. Reproduced with permission.^24^ Copyright 2023, Wiley. (D) A schematic illustration of the fabrication process for a vertical TEG using flexible thermoelectric films.^153^ Copyright 2025, Elsevier. (E) The schematic diagram of the preparation process and transport measurements of Cu-intercalated samples.^154^ Copyright 2022, Wiley. (F) Thermoelectric generator applications with multifunctionality and efficient heat collection.^155^ Copyright 2020, Elsevier.

As shown in Figure 2A, Dong Yang et al.^151^ explored the potential of Ag_2_Se for wearable thermoelectric applications. In this study, n-type Ag_2_Se and p-type Sb_2_Te_3_ were employed as thermoelectric electrodes, with Cu used as the interconnection material. The device was fabricated by vacuum thermal co-evaporation. The authors focused on Te doping in Ag_2_Se to enhance its thermoelectric performance, achieving a ZT value as high as 1.27 at 363 K. A single p-n thermocouple was evaluated, yielding a power density of 1.5 mWcm^−2^, an open-circuit voltage of 6 mV, and an output power of 65 nW under a temperature difference of 20 K. Through Te-doping-induced (00l) orientation engineering, high-performance Ag_2_Se thermoelectric films were fabricated on flexible polyimide (PI) substrates, providing a material and structural foundation for low-temperature flexible TEGs.

In parallel with inorganic-film optimization, organic and hybrid thermoelectric systems have been investigated to improve stretchability and conformability. Although their thermoelectric performance is generally lower than that of inorganic counterparts, their mechanical compliance enables device operation under repeated bending, stretching, and attachment to irregular surfaces. As shown in Figure 2B, Mohamed Ali et al.^152^ reported a flexible and stretchable organic TEG employing p-type poly(3,4-ethylenedioxythiophene):poly(styrene sulfonate) (PEDOT:PSS) and n-type single-walled carbon nanotubes single-walled carbon nanotubes (SWCNTs) as thermoelectric electrodes, with Cu interconnects patterned in a serpentine geometry. The device was encapsulated to enhance mechanical stability, while the serpentine interconnect design imparted improved stretchability to the TEG. Thin films were fabricated using laser cutting, 3D-printed molds, and drop-casting techniques. To improve electrical conductivity, 5% DMSO was added to the p-type electrode, and SWCNT films were doped with polyethyleneimine (PEI) to achieve n-type conduction. A vertically stacked TEG comprising four thermocouple pairs was fabricated. Under a temperature difference of 29.4°C, a maximum output power of 887.5 nW was obtained, while a power density of 216 nWcm^−2^ was achieved at a temperature difference of 50°C. This work demonstrated comparatively high output power and excellent stretchability among organic-material-based TEGs, providing useful design guidelines for wearable flexible devices.

Device architecture is equally important because the temperature difference actually established across the thermoelectric legs may be substantially smaller than the temperature difference between the heat source and the surrounding environment. Textile-supported, self-supporting, and vertically stacked structures have, therefore, been developed to reduce parasitic heat loss and improve the utilization of the available temperature gradient. Kim et al.^156^ reported a wearable glass-fabric-based TEG designed to mitigate the severe thermal losses commonly associated with ceramic substrates used in inorganic thermoelectric devices. By employing glass fibers combined with a self-supporting architecture, this work enabled direct printing of thermoelectric materials onto glass fibers. Screen-printed Bi_2_Te_3_ (n-type) and Sb_2_Te_3_ (p-type) electrodes were fabricated on the glass fibers, while Cu embedded in PDMS served as the interconnection electrodes. A bandage-shaped flexible TEG consisting of 11 thermocouple pairs was designed and applied to human skin for thermal energy harvesting. The device generated an output voltage of 2.9 mV and delivered an output power of 3 μW under a matched load at an ambient temperature of 15°C. Under a temperature difference in the range of 300–350 K, a power density of 3.8 mWcm^−2^ and a specific power of 28 mWg^−1^ were achieved. As illustrated in Figure 2D, Jongin Won et al.^153^ improved the thermoelectric performance of Ag_2_Se by introducing trace amounts of Cu_2_Se nanoparticles. This strategy increased the ZT value to 0.55 at room temperature. In addition, a vertically stacked TEG architecture was proposed to more efficiently exploit the temperature gradient across the human body for power generation. A device comprising 20 vertically stacked thermocouples was fabricated and tested, achieving a power density of 2.6 μWcm^−2^ under a temperature difference of 10 K. Notably, this substrate-free, self-supporting structure reduced reliance on rigid substrates and significantly enhanced mechanical flexibility.

Material-level optimization of Bi_2_Te_3_-based compounds has focused on simultaneously regulating carrier transport, phonon scattering, and mechanical integrity. Interfacial precipitates, modulation doping, elemental intercalation, and secondary-phase incorporation have been employed to improve the power factor or reduce lattice thermal conductivity while maintaining processability. To enhance the thermoelectric performance of Bi_0_._5_Sb_1_._5_Te_3_, Deyu Bao et al.^157^ proposed an interfacial engineering strategy based on amorphous Sb_2_S_3_. During sintering, amorphous Sb_2_S_3_ decomposed into metallic Sb nanoprecipitates and S dopant atoms, forming semi-coherent interfaces. The precipitated Sb, in conjunction with the Bi_0_._5_Sb_1_._5_Te_3_ matrix, induced an energy-filtering effect that enhanced the Seebeck coefficient. Meanwhile, S dopants increased the carrier concentration, while the introduced Sb generated multiscale defects that promoted phonon scattering, thereby reducing the lattice thermal conductivity. As a result, a peak ZT value of 1.31 was achieved at 330 K. Under a temperature difference of 180 K, a power density of approximately 0.15 Wcm^−2^ and an output power of 24 mW were obtained. This work demonstrates synergistic optimization of electrical and thermal transport properties without compromising electrical conductivity, providing an effective strategy for low-temperature thermoelectric material optimization.

As shown in Figure 2E, Cheng-Lung Chen et al.^154^ addressed the long-standing mismatch between n-type and p-type thermoelectric performance in Bi_2_Te_3_-based materials by focusing on improving the ZT of n-type compounds. In their research, CuI doping was introduced into Bi_2_Te_2_._7_Se_0_._3_, followed by Cu intercalation into (CuI)_0_._002_Bi_2_Te_2_._7_Se_0_._3_. These combined strategies enhanced electron mobility while forming pseudo-superlattice interfaces that strongly scattered phonons, leading to reduced lattice thermal conductivity. A power factor of 63.5 μWcm^−1^K^−2^ was achieved at 300 K with 0.2% Cu intercalation, along with a peak ZT of 1.42 and an average ZT of 1.36 over the temperature range of 300–450 K. This work established a new performance benchmark for n-type Bi_2_Te_3_-based thermoelectric materials and significantly alleviated the long-standing performance limitations of n-type thermoelectrics.

Bin Chen et al.^158^ introduced MgB_2_ doping into Bi_2_Te_2_._7_Se_0_._3_ to simultaneously enhance its thermoelectric performance, mechanical hardness, and machinability. The doped material was fabricated via a one-step spark plasma sintering (SPS) process, enabling scalable production. An average ZT value of 0.88 was achieved over the temperature range of 300–500 K, accompanied by a hardness of 0.60–0.67 GPa, significantly exceeding that of commercial materials. The authors further demonstrated that excessive doping led to ZT degradation, identifying 0.5% MgB_2_ as the optimal concentration. At 300 K, the material exhibited a power factor of 3.3 mWm^−1^K^−2^. Its hardness increased by approximately 70%, cracks were nearly eliminated, and machinability was significantly enhanced. This work provides a promising pathway for the scalable, low-cost fabrication of high-performance n-type Bi_2_Te_3_-based alloys.

Beyond conventional Bi2Te3-based compounds, thin-film materials with low thermal conductivity have been explored for planar and flexible generators. These studies emphasize the compatibility of the material with deposition, patterning, and mechanically compliant interconnects rather than with the peak ZT value alone. Chenjin Liu et al.^159^ developed thermoelectric materials exhibiting both low thermal conductivity and high ZT. In this study, amorphous FeVSb-based thin films were fabricated for the first time, and Ti doping enabled a transition from n-type to p-type conduction. FeVSb-based HH alloys are recognized for their high thermoelectric potential, low cost, and environment friendliness and can be tuned between n-type and p-type through doping. Based on n-type FeVSb and p-type (FeVSb)_0_._94_Ti_0_._06_ films, a planar TEG comprising four thermocouple pairs was fabricated. At 300 K, ZT values of 0.09 and 0.23 were obtained for FeVSb and (FeVSb)_0_._94_Ti_0_._06_, respectively. Under a temperature difference of 59.4 K, a maximum output power of 248 nW was achieved. Notably, the thermal conductivities of FeVSb and (FeVSb)_0_._94_Ti_0_._06_ were as low as 1.18 and 0.93 Wm^−1^K^−1^, respectively, both significantly lower than those of conventional HH alloys, demonstrating the potential of amorphous thin-film TEGs. As shown in Figure 2F, Vaithinathan Karthikeyan et al.^155^ addressed the limitations of conventional TEGs for wearable applications by emphasizing flexibility, thin-film integration, and high power output. p-Type SnTe and n-type PbTe were selected as thermoelectric materials, exhibiting ZT values of 0.3 and 0.2 at 550 K, respectively. Devices were fabricated via thermal evaporation using metal shadow masks. Under a temperature difference of 120 K, a power density of 8.4 mWcm^−2^ was achieved. Owing to the serpentine structural design, the device maintained stable performance after 400 bending cycles. When worn on the wrist, an output voltage of 5.3 mV was generated under a temperature difference of 10 K, sufficient to power a small light-emitting device. This study simultaneously addresses mechanical durability and multi-scenario energy harvesting, providing a scalable approach for battery-free electronic skin and industrial waste-heat recovery.

Secondary-phase engineering and conductivity-type regulation have also enabled low-temperature thermoelectric devices to combine energy harvesting with additional functions such as temperature sensing. However, the practical value of these approaches should be evaluated using device-level output, mechanical durability, and long-term interface stability in addition to material-level ZT.

Tao Chen et al.^160^ investigated the inferior low-temperature thermoelectric performance of n-type Bi_2_Te_2_._7_Se_0_._3_ (BTS) compared with p-type counterparts. Ag_9_AlSe_6_ nanoparticles were introduced as a secondary phase to act as phonon-scattering centers and reduce thermal conductivity. By optimizing the Ag_9_AlSe_6_ content, a 43% reduction in thermal conductivity was achieved at BTS-0.35 vol % Ag_9_AlSe_6_. The maximum ZT reached 1.2 at 423 K, with an average ZT of 1.1 over the temperature range of 300–473 K, representing a 29% improvement over pristine BTS. By introducing Ag_9_AlSe_6_ nanoparticles, this approach effectively enhanced thermoelectric performance by simultaneously reducing lattice thermal conductivity and carrier concentration. Jian Wang et al.^161^ improved the thermoelectric performance of Sn-doped Bi_2_Se_3_ by inducing a transition from n-type to p-type conduction, with 3% Sn identified as the optimal doping level. To further enhance sensitivity, Bi_1_._97_Sn_0_._03_Se_3_ was composited with PEDOT:PSS and fabricated on PET substrates via screen printing, improving electrical conductivity. A dual-chain TEG composed of n-type Bi_2_Se_3_ and p-type Bi_1_._97_Sn_0_._03_Se_3_ was constructed, achieving a sensitivity of −0.63%/°C and a temperature resolution of 0.2°C. Under a temperature difference of 70 K, an output voltage of 76.06 mV was obtained. This study demonstrates the potential of thermoelectric devices for simultaneous energy harvesting and self-powered temperature sensing, featuring high sensitivity and providing a novel strategy for further enhancing their sensing performance in future research.

Overall, Bi_2_Te_3_- and Ag_2_Se-based systems currently provide the strongest combination of near-room-temperature material performance and device-level validation. Improvements based on carrier regulation, orientation control, and secondary-phase engineering have repeatedly increased the power factor or reduced lattice thermal conductivity. Their practical value is most convincing when the material improvement is accompanied by complete-device output measurements, stable contacts, and mechanical testing. Organic and hybrid thermoelectric systems are strategically valuable for stretchability, conformability, and low-cost processing, but their output density and long-term environmental stability generally remain less established than those of inorganic systems. Similarly, material studies reporting only a high peak zT should be regarded as evidence of material potential, rather than mature wearable-generator technology. Further progress is, therefore, more likely to result from improved heat-source coupling, low-resistance interfaces, mechanical durability, and power management than from isolated increases in peak ZT. Representative low-temperature thermoelectric materials, fabrication routes, and device-performance metrics are summarized in Table 1.

**Table 1:** Low-temperature thermoelectric generators

Thermoelectric material (n/p)	Fabrication process	ZT value	Power density	Output power	Power factor	Operating Temperature Range
Ag_2_Se/Sb_2_Te_3_^151^	Vacuum thermal co-evaporation	1.27 (363 K)	1.5 mWcm^−2^ (ΔT = 20 K)	65 nW (ΔT = 20 K)	24.8 μWcm^−1^K^−2^ (363 K)	300–363 K
Bi_0_._5_Sb_1_._5_Te_3_^157^	Spark plasma sintering	1.31 (330 K)	0.15 Wcm^−2^ (ΔT = 180 K)	24 mW (ΔT = 180 K)	3345.40 μWm^−1^K^−2^ (330 K)	300–480 K
Bi_2_Te_2_._7_Se_0_._3_-0.5%MgB_2_^158^	Melting–quenching → grinding → single-step SPS	0.96 (325 K)	–	–	3.3 mWm^−1^ K^−2^ (300 K)	300–500 K
(FeVSb)_0_._94_Ti_0_._06_^159^	Magnetron sputtering	0.23 (300 K)	–	248 nW (ΔT = 59.4 K)	0.70–1.20 mWm^−1^K^−2^ (300–475 K)	300–473 K
Bi_2_Te_2_._7_Se_0_._3_-0.35vol%Ag_9_AlSe_6_^160^	Vacuum melting → quenching → annealing; hand grinding + oscillatory ball milling; hot pressing	1.2 (423 K)	–	–	29.3 μWcm^−1^K^−2^ (300 K)	300–473 K
Ag_2_Se + Cu_2_Se nanoparticle doping^153^	Ink formulation → drop casting → hot pressing → peeling → annealing	0.55 (300 K)	2.59 μWcm^−2^ (ΔT = 10 K)	–	1,680 ± 120 μWm^−1^K^−2^ (300 K)	293–300 K
(CuI)_0_._002_Bi_2_Te_2_._7_Se_0_._3_ + 0.2%Cu^154^	Thermally driven method	1.42 (375 K)	–	–	63.5 μWcm^−1^K^−2^ (300 K)	300–450 K
Bi_2_Te_3_^162^	Two-step single-source thermal evaporation + rapid thermal annealing	0.51 (400 K)	–	–	0.53 mWm^−1^ K^−2^ (478 K)	300–483 K
Ag_2_Se + 5%wt Te^163^	Screen printing + SPS	1.06 (303 K)	1.9 mWcm^−2^ (ΔT = 20 K)	26.2 μW (ΔT = 20 K)	25.7 μWcm^−1^K^−2^ (303 K)	303–383 K
Bi_2_Te_2_._7_Se_0_._3_ nanosheets^164^	Microwave-assisted solvothermal reaction	1.23 (480 K)	–	–	19 × 10^−4^ Wm^−1^K^−2^ (440 K)	300–500 K

### Medium-temperature thermoelectric materials and generators

Medium-temperature thermoelectric materials are mainly developed for automotive exhaust, boilers, and industrial waste-heat sources.^165^^,^^166^^,^^167^^,^^168^^,^^169^^,^^170^^,^^171^^,^^172^^,^^173^^,^^174^^,^^175^ The principal material target is a high average zT over a broad operating range, while practical modules additionally require chemically stable interfaces, low contact resistance, compatible p-type and n-type legs, and scalable fabrication. Accordingly, this section first discusses transport-property optimization and then examines representative interface- and module-level demonstrations.

**Figure 3:**
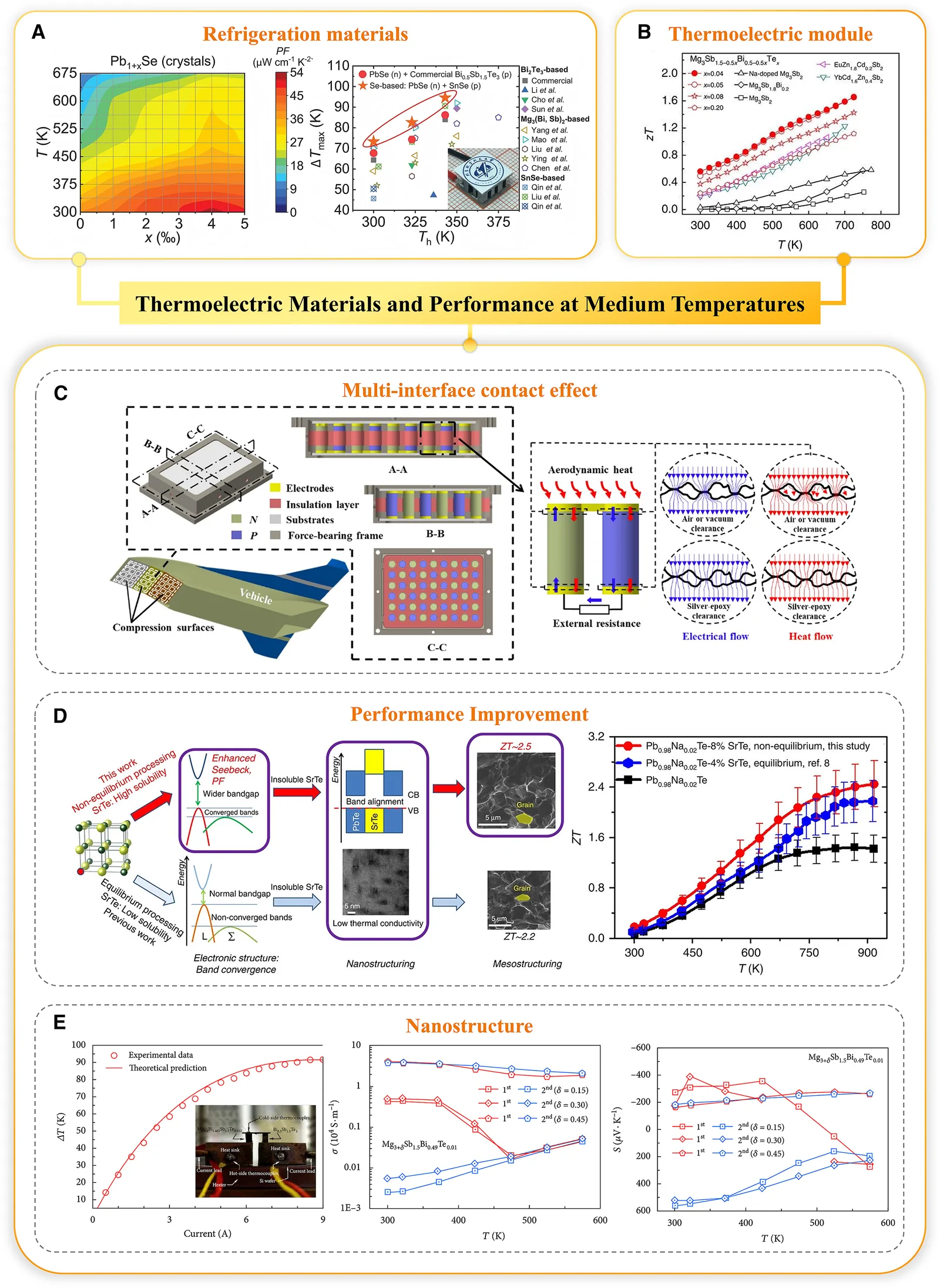
Medium-temperature thermoelectric generators (A) Grid-plainification strategy by manipulating composition.^176^ Copyright 2024, AAAS. (B) ZT value of n-type Mg_3_Sb_2_-based Zintl compound.^177^ Copyright 2017, Springer Nature. (C) Schematic diagram of TEGs for a high-speed flight vehicle.^178^ Copyright 2025, Elsevier. (D) Roadmap toward record-high thermoelectric performance in Pb_0.98_Na_0.02_Te-x%SrTe polycrystals by non-equilibrium synthesis.^179^ Copyright 2016, Springer Nature. The scale bars are 5 nm for the nanostructuring image and 5 μm for the mesostructuring images. The ZT plot includes 15% error bars, as reported in the original study. (E) Electrical current dependence of temperature difference between the hot and cold sides at the hot-side T of 350 K.^180^ Copyright 2020, AAAS.

Medium-temperature material development has primarily focused on maintaining a high average ZT over a broad temperature range. Carrier-concentration optimization, band convergence, defect control, and multiscale phonon scattering are the principal strategies used to improve electrical and thermal transport simultaneously. As shown in Figure 3A, Yongxin Qin et al.^176^ developed a Te-free PbSe-based thermoelectric system aimed at high-performance room-temperature cooling applications. The thermoelectric performance was enhanced through a combination of grid-like compositional design and defect engineering. Trace Cu doping supplied electrons, while excess Pb compensated for Pb vacancies, effectively optimizing the material for cooling applications. The Pb_1_._004_Se sample exhibited a ZT of 0.9 at room temperature, with a power factor above 52 μW cm^−1^ K^−2^ and a record-high carrier mobility of 2,200 cm^2^ V^−1^ s^−1^. This enhancement is primarily attributed to the defect-filling strategy, which significantly improved the thermoelectric properties of PbSe. Consequently, the device achieved a maximum cooling temperature difference of 73.3 K and a power generation efficiency of 11.2% under a 420 K temperature gradient. These results provide a scalable pathway for the development of next-generation sustainable thermoelectric cooling technologies. As shown in Figure 3B, Jiawei Zhang et al.^177^ investigated a novel thermoelectric material to enhance performance. Te was doped into Mg_3_Sb_1_._5_Bi_0_._5_ via SPS. The resulting sample exhibited ZT values of 0.56–1.65 across 300–725 K, surpassing many existing thermoelectric materials. At room temperature, the sample achieved a power factor of 13.9 μW cm^−1^ K^−2^ and its Seebeck coefficient reached 268 μV K^−1^ at 725 K. Moreover, the thermal conductivity decreased to 0.556 W m^−1^ K^−1^ at 725 K, significantly enhancing ZT. This improvement is attributed to the sample’s distinctive multi-valley conduction band structure, which markedly boosts both the power factor and ZT, demonstrating its potential for medium-temperature thermoelectric applications.

Translating these material-level improvements into generator performance requires stable electrical and thermal interfaces. Diffusion-barrier selection, contact-medium optimization, and control of interfacial reactions are, therefore, essential for limiting contact resistance and preserving performance during high-temperature exposure. Tong Xing et al.^181^ investigated strategies to enhance the thermoelectric performance of GeTe-based materials. The sample, co-doped with Sb, Bi, and Se, achieved a ZT of 2.0 at 700 K. To prevent high-temperature reactions between GeTe and common electrode materials, a diffusion barrier layer was introduced. Screening of 12 pure metals identified Mo as the least reactive with GeTe, which was consequently chosen as the barrier material. Under a 500 K temperature gradient, the Ge_0_._92_Sb_0_._04_Bi_0_._04_Te_0_._95_Se_0_._05_ sample delivered a power density of 1.1 W cm^−2^, while the Mo-GeTe interface resistance remained below 1 mΩ cm^2^, ensuring excellent thermal stability. As a result, the energy conversion efficiency reached 7.8%, providing a practical benchmark for the application of GeTe-based thermoelectric materials.^182^ As shown in Figure 3C, ShiYuan Chen et al.^178^ studied the influence of multi-interface contacts on TEGs, developing numerical models for TCR and ECR to replace microscale rough surface models. This approach reduced computational complexity while maintaining high accuracy. TCR and ECR were further measured using different contact media, with silver epoxy yielding the lowest resistance values. The TEG achieved a maximum output voltage of 6.978 V and a peak output power of 12.41 W. Optimization of the contact medium and increased contact pressure significantly reduced TCR and ECR, thereby improving the thermoelectric performance. The equivalent interface layer model also demonstrated excellent performance in aerodynamic heat recovery for high-speed aircraft, providing guidance for the exploration of other high-thermal-conductivity, low-resistance contact materials.

Yingcai Zhu et al.^183^ investigated strategies to enhance the thermoelectric performance of PbSe by employing band engineering. For the first time, the Λ valence band (Nv = 8) was activated in p-type PbSe, achieving triple valence band convergence. The introduction of AgInSe_2_ nanoprecipitates along with a high density of dislocations reduced the lattice thermal conductivity to 0.31 W m^−1^ K^−1^ at 873 K. A composite material was constructed using 2% Na doping combined with AgInSe_2_. The Pb_0_._98_Na_0_._02_Se-2.05% AgInSe_2_ sample exhibited a power factor of 15.6 μW cm^−1^ K^−2^ at 423 K and a peak ZT of 1.9 at 873 K. AgInSe_2_ doping significantly increased the effective mass without substantially affecting carrier mobility. Furthermore, the energy differences between the L and Σ valence bands (ΔE_1-2_) and between the L and Λ valence bands (ΔE_1-3_) decreased with increasing temperature, promoting a higher Seebeck coefficient and further enhancing thermoelectric performance. Ananya Banik et al.^184^ addressed the limitations of intrinsic SnTe, where Sn vacancies led to excessively high hole concentrations, resulting in low Seebeck coefficients and ZT values. They implemented a strategy combining Sn self-compensation with Mg alloying to enhance thermoelectric performance. Sn self-compensation first suppressed hole concentration, followed by Mg alloying to further engineer the band structure. Mg alloying reduced the energy difference ΔE to approximately 0.18 eV, achieving valence band convergence. The approach also expanded the bandgap (0.11 → 0.26 eV), reduced the L-Σ valence band separation (0.35 → 0.18 eV), and increased the effective mass m∗ (0.16 → 0.69 m_e_). The p-type Sn_0_._94_Mg_0_._09_Te sample achieved a ZT of 1.2 at 856 K, with a Seebeck coefficient of 175 μV K^−1^, thermal conductivity of 2.2 W m^−1^ K^−1^, and a power factor of 30.3 μW cm^−1^ K^−2^, placing it among the highest-performing SnTe-based materials. Importantly, the sample could be synthesized via a straightforward vacuum melting process, demonstrating excellent practical scalability. Yu Xiao et al.^185^ investigated the long-standing issue of n-type PbTe exhibiting lower ZT values compared with its p-type counterpart. Cu doping was employed to achieve multiple functions: compensating Pb vacancies and, at higher concentrations, forming Cu_2_Te precipitates that induced multi-scale phonon scattering, thereby reducing lattice thermal conductivity to 0.38 W m^−1^ K^−1^. The introduced Cu had minimal impact on the effective mass, ensuring that the enhancement in power factor primarily resulted from increased carrier concentration rather than a sacrifice in Seebeck coefficient. The material was synthesized via a melt followed by SPS sintering. The PbTe-2% Cu_2_Te sample exhibited an average ZT of 1.0 over 300–873 K, a peak power factor of 36.7 μW cm^−1^ K^−2^, and a carrier mobility of 1,090 cm^2^ V^−1^ s^−1^. This study is the first to demonstrate a multilevel cooperative strategy within a single system, simultaneously enhancing carrier mobility and reducing lattice thermal conductivity, thereby elevating n-type PbTe performance to be comparable with its p-type counterpart.

As shown in Figure 3D, Gangjian Tan et al.^179^ achieved a non-equilibrium thermoelectric breakthrough in the PbTe-SrTe system by doping SrTe into PbTe, enabling stable operation across 300–923 K. The experimental sample, Pb_0_._98_Na_0_._02_Te-8% SrTe, utilized Na to compensate for Pb vacancies. Under a temperature of 923 K, the sample exhibited a power factor of 22 μW cm^−1^ K^−2^, with a lattice thermal conductivity reduced to 0.5 W m^−1^ K^−1^, resulting in an average ZT of 1.67 across the 300–900 K temperature range. By employing a non-equilibrium quenching-annealing strategy, the solubility of SrTe was increased from <1% to 5%. This work represents the first demonstration in bulk PbTe of the simultaneous realization of band convergence, full-scale phonon scattering, and band alignment, offering a viable pathway for medium-temperature waste-heat recovery and hotspot energy harvesting. ZhongZhen Luo et al.^186^ investigated strategies to enhance the thermoelectric performance of n-type PbTe. By introducing a trace amount of Zn into Ga-doped PbTe, the sample achieved an average ZT of 1.26 over the 400–873 K range. The study revealed that Zn induced the precipitation of Ga_2_Te_3_ secondary phases and generated Te vacancies, which increased the carrier concentration. Remarkably, less than 1% Zn was sufficient to reduce the lattice thermal conductivity by approximately 26%. The optimized sample, Pb_0_._975_Ga_0_._025_Te-0.25% ZnTe, was fabricated via vacuum melting and SPS, showing significant performance improvements over comparable n-type thermoelectric materials and achieving a ZT exceeding 1.2 for the first time. This study offers an effective strategy for future thermoelectric materials research.

Module-level studies provide a more direct assessment of practical performance because they incorporate p-n material matching, contact resistance, electrode design, and heat-transfer losses. Segmented modules are particularly attractive in this temperature regime because different material systems can be assigned to temperature intervals in which they exhibit their highest efficiency. As shown in Figure 3E, Xiaokai Hu et al.^187^ first applied nanostructured PbTe in thermoelectric modules and introduced a diffusion barrier layer to reduce contact resistance. Nanoprecipitation and doping were employed to enhance ZT, with PbTe-2%MgTe-4%Na as the p-type material and PbTe-0.2%PbI_2_ as the n-type material, where nanoprecipitates scattered phonons. Modules were fabricated through vacuum melting, ball milling, and SPS, incorporating a Co_0_._8_Fe_0_._2_ diffusion barrier layer to suppress elemental diffusion and minimize interfacial resistance. The module reached a ZT of 1.8 at 810 K. Building on this, a segmented module combining low-temperature Bi_2_Te_3_ and mid-temperature nanostructured PbTe was designed, achieving a power density of 1.2 W cm^−2^, a maximum output of 2.34 W, and an efficiency of 11% under a 590 K temperature difference. This study translates the high ZT of nanostructured PbTe into module-level performance through innovations in materials, interfaces, and structure, offering a viable strategy for waste-heat recovery applications.

Band convergence, carrier-concentration regulation, defect engineering, and hierarchical phonon scattering have been repeatedly successful in improving the material-level performance of PbTe-, PbSe-, SnTe-, GeTe-, and Mg_3_Sb_2_-based systems. However, the strongest evidence of practical progress is provided by studies that combine high average ZT with low-resistance diffusion barriers, compatible p-type and n-type legs, and segmented module architectures. These studies demonstrate that interface and module design can determine whether material improvements are retained at the generator level. Approaches supported only by peak ZT values, without contact-resistance, conversion-efficiency, or thermal-cycling data, should still be regarded as promising material concepts rather than mature generator technologies. The most important remaining limitations are long-term interfacial stability, the use of Pb- and Te-containing compositions, scalable densification, and maintenance of the effective module temperature difference under realistic heat-source conditions. Representative medium-temperature thermoelectric materials, processing routes, and performance metrics are summarized in Table 2.

**Table 2:** Medium-temperature thermoelectric generators

Thermoelectric material (n/p)	Fabrication process	ZT value	Power density	Output power	Power factor	Operating Temperature Range
Mg_3_Sb_1_._5_Bi_0_._5_^177^	Arc melting and SPS	1.65 (725 K)	–	–	14.14 μWcm^−1^K^−2^ (525 K)	300–725 K
Pb_0_․_98_Na_0_․_02_Se-2.05%AgInSe_2_^183^	Vacuum melting; ball milling; SPS	1.9 (873 K)	–	–	15.6 μWcm^−1^K^−2^ (423 K)	300–873 K
p–type Sn_0_._94_Mg_0_._09_Te^184^	Melting synthesis	1.2 (856 K)	–	–	30.3 μWcm^−1^ K^−2^ (856 K)	300–873 K
PbTe-2 %Cu_2_Te^185^	Melting and ingot casting; SPS	1.5 (723 K)	–	–	36.7 μWcm^−1^K^−2^ (423 K)	300–873 K
Pb_0_._975_Ga_0_._025_Te–0.5% ZnTe^186^	Vacuum melting; SPS	1.55 (723 K)	–	–	35 μWcm^−1^K^−2^ (373 K)	300–873 K
Ge_0_._92_Sb_0_._04_Bi_0_._04_Te_0_._95_Se_0_._05_^181^	Melting method + SPS	2.0 (700 K)	1.1Wcm^−2^(ΔT = 500 K)	2.0W(ΔT = 500 K, 8 pairs)	–	300–800 K
Pb_1_._004_Se^176^	Physical vapor deposition with excess-Pb self-compensation	1.8 (623 K)	–	59 mW (ΔT = 420 K)	52 μWcm^−1^K^−2^ (300 K)	300–673 K
Pb_0_._98_Na_0_._02_Te-8%SrTe^179^	High-temperature melting + rapid quenching + intermediate-temperature annealing + SPS densification	2.5 (923 K)	–	–	30 μWcm^−1^K^−2^ (500 K)	300–923 K
Mg_3_Sb_1_._5_Bi_0_._49_Te_0_._01_^180^	Ball milling + hot pressing	1.5 (700 K)	–	–	–	300–700 K
(Ti_0_._33_Zr_0_._33_Hf_0_._33_Al_0_._005_Sc_0_._005_)NiSn^188^	Arc melting + hot pressing	0.77 (750 K)	–	–	5.2 μWcm^−1^K^−2^ (750 K)	300–800 K

### High-temperature thermoelectric materials and generators

High-temperature TEGs are primarily applied in aerospace,^189^^,^^190^^,^^191^ metallurgy, and gas turbines,^192^^,^^193^ where operations occur under very high temperatures, thereby requiring high-temperature TEGs to perform reliably. Research on high-temperature TEGs is still relatively limited, making studies in this area both necessary and promising. Its core application scenarios include ultra-high temperature industrial kiln waste-heat recovery in metallurgy, cement, glass industry, aerospace, concentrated-solar thermoelectric power generation, hypersonic aircraft aerodynamic heat recovery, etc. This temperature regime contains high-energy-density waste-heat sources and is, therefore, important for industrial energy recovery and is also an attractive solid-state power-generation option for extreme environments such as aerospace. The core constraints are the intensification of intrinsic excitation and bipolar diffusion effects at high temperatures, the serious volatilization of elements, and the sharp amplification of interfacial diffusion and thermal stress mismatches.

**Figure 4:**
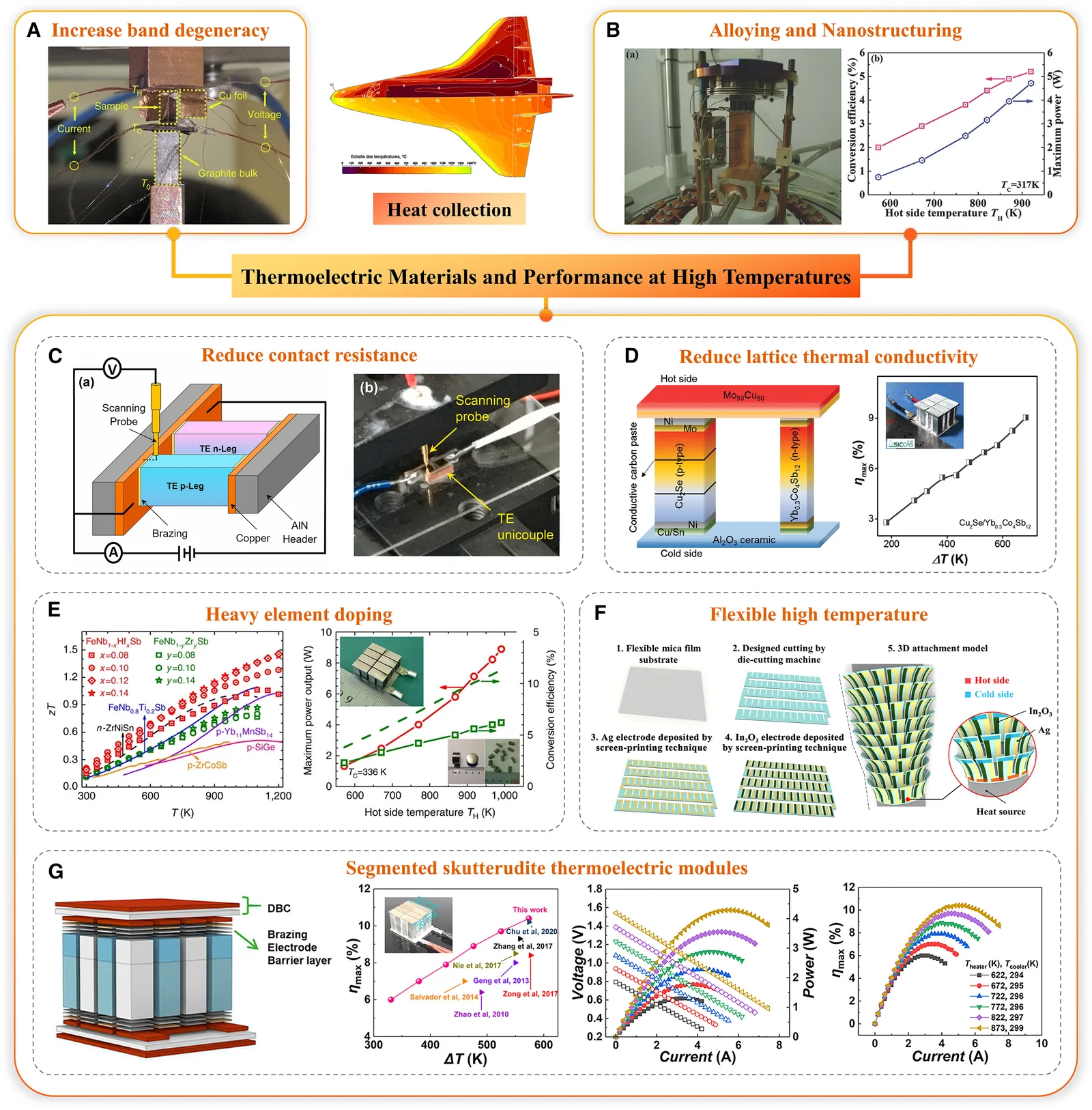
High-temperature thermoelectric generators (A) Measurement of output power density and thermoelectric conversion efficiency.^194^ Copyright 2018, Springer Nature. (B) ZrNiSn/FeNbTiSb-based half-Heusler thermoelectric module performance characterization.^195^ Copyright 2015, Wiley. (C) Four-probe contact resistance measurement system and results.^196^ Copyright 2021, Elsevier. (D) Temperature-dependent lattice thermal conductivity (κ_l_) and dimensionless figure of merit of the mosaic-structured Cu_2_X in comparison with the pristine Cu_2_Se.^197^ Copyright 2020, Wiley. (E) Thermoelectric performance for p-type FeNbSb-based HH compounds and prototype module.^198^ Copyright 2015, Springer Nature. (F) Preparation process of >1,000°C F-TEGs.^199^ Copyright 2023, Springer Nature. (G) Development of segmented skutterudite thermoelectric modules for higher efficiency.^200^ Copyright 2023, Elsevier.

HH alloys constitute one of the most extensively investigated high-temperature thermoelectric material families because of their high power factors, mechanical robustness, and comparatively good phase stability. Current material-level strategies mainly include band-degeneracy enhancement, carrier-concentration regulation, heavy-element alloying, and multiscale phonon scattering. As shown in Figure 4A, Hangtian Zhu et al.^194^ investigated strategies to enhance the thermoelectric performance of HH materials, focusing on ZrCoBi-based compounds. In ZrCoBi, the valence bands at the Γ and L points nearly converge in energy, resulting in a high band degeneracy of 10, thereby significantly enhancing the power factor. The material also exhibits a low average sound velocity, leading to reduced lattice thermal conductivity. Further reduction in lattice thermal conductivity was achieved through Sn doping, and subsequent Sb doping in ZrCoBi_0_._65_Sb_0_._15_Sn_0_._2_ lowered it even further, culminating in a peak ZT of approximately 1.42 at 973 K. The material demonstrated a power density of 9.3 W cm^−2^ at 823 K and a room temperature power factor of 40 μW cm^−1^ K^−2^. Its combination of excellent thermal stability and low thermal conductivity underscores its strong potential for high-temperature thermoelectric applications. The development of HH materials such as this provides new insights for future thermoelectric material research. Hangtian Zhu et al.^201^ applied a reverse-design strategy to discover and optimize new HH thermoelectric materials, selecting TaFeSb-based compounds. By doping with Ti and V, the Ta_0_._74_V_0_._1_Ti_0_._16_FeSb sample achieved an average ZT of 0.93 over the 300–973 K range, with a maximum conversion efficiency of 11.4% and a room-temperature power factor of 56 μW cm^−1^ K^−2^. The material was prepared via a two-step ball milling and hot-pressing process. Theoretical calculations of the electronic structure revealed a unique band configuration, with the valence band maximum located at the L point and two converging valence bands providing a high valley degeneracy (Nv = 8). This high degeneracy contributes to the large power factor, while Ti doping further optimized the thermoelectric properties by significantly increasing electrical conductivity and reducing the Seebeck coefficient. The reverse-engineering approach employed in this study offers a valuable method for discovering additional promising materials for TEG applications.

Ran He et al.^202^ investigated methods to enhance the performance of thermoelectric materials by increasing the power factor. While most studies focus on reducing thermal conductivity to improve thermoelectric performance, this work employed higher hot-pressing temperatures to enhance the power factor of p-type HH Nb_0_._95_Ti_0_._05_FeSb to approximately 106 μW cm^−1^ K^−2^. Based on this high power factor, a single-leg device achieved an output power density of around 22 W cm^−2^ across 293–868 K. The samples were prepared using arc melting, ball milling, and hot pressing, with Nb_0_._95_Ti_0_._05_FeSb treated at various hot-pressing temperatures, yielding the optimal sample at 1373 K. Additionally, ZT was further optimized through Ti doping, and the Nb_0_._8_Ti_0_._2_FeSb sample reached a ZT of 1.1 at 973 K, demonstrating excellent high-temperature performance and providing an effective strategy for enhancing thermoelectric properties in high-temperature TEGs. As shown in Figure 4B, Tiejun Zhu et al.^195^ developed high-performance HH thermoelectric materials to overcome the typical limitations of thermal stability and mechanical strength in high-temperature applications, achieving relatively high energy conversion efficiency. They introduced a novel p-type Fe(V,Nb)Sb-based material and performed microstructural analyses, revealing that Ni atoms occupying vacancies generate additional electronic states, which affect charge carrier transport. The materials were fabricated using levitation melting followed by SPS sintering. For testing, the HH thermoelectric module incorporated n-type ZrNiSn-based material and p-type FeNb_0_._8_Ti_0_._2_Sb. The p-type material exhibited a ZT of 1.1 at 1,100 K, with a power factor of approximately 3.0 × 10^−3^ W m^−1^ K^−2^ at 900 K, while the n-type material achieved a ZT of 1.0 and a power factor of about 4.5 × 10^−3^ W m^−1^ K^−2^ at 900 K. This study demonstrates that rapid fabrication, alloying, and nanostructuring are effective strategies to optimize the thermoelectric performance of HH materials, providing valuable guidance for future research.

For HH materials, module performance is strongly influenced by contact formation and interfacial stability. High material-level ZT cannot be fully translated into generator efficiency when metallization layers, solders, or diffusion barriers introduce excessive electrical resistance or undergo interfacial reactions during high-temperature operation. Figure 4C illustrates the strategy proposed by Amin Nozariasbmarz et al.^196^ to reduce contact resistance in high-temperature thermoelectric devices, addressing the limitations of conventional approaches that require prior metallization, which typically results in elevated resistance. In this study, a one-step forming technique was employed, wherein Cu-Ag solder directly wetted the HH legs without prior metallization treatment. The barrier layer was simultaneously upgraded using high-temperature Cu-Ag solder, which forms a diffusion layer with both n- and p-type materials, thereby ensuring mechanical strength and high electrical conductivity. For experimental validation, p-type Hf_0_._5_Zr_0_._5_CoSb_0_._8_Sn_0_._2_ and n-type Hf_0_._75_Zr_0_._25_NiSn_0_._99_Sb_0_._01_ were selected. The module demonstrated an output power density of 11.5 W cm^−2^ at 670 K, a conversion efficiency of 9.5%, and a contact resistance below 1 μΩ cm^2^. This approach of directly wetting HH surfaces with Cu-Ag solder, without prior metallization, demonstrates an effective strategy for minimizing contact resistance in high-temperature thermoelectric devices.

Building on the material-, module-, and contact-level studies discussed above, recent generator-level investigations have clarified the engineering route required to translate HH performance into practical electrical output. Xing et al. employed self-propagating high-temperature synthesis to prepare approximately 200-g batches of n-type Zr_0.5_Hf_0.5_NiSn_0.985_Sb_0.015_ and p-type Zr_0.5_Hf_0.5_CoSb_0.8_Sn_0.2_ alloys while retaining thermoelectric properties comparable to those of laboratory-scale samples.^72^ Three-dimensional finite-element optimization of the leg dimensions, area ratio, fill factor, and parasitic heat losses subsequently enabled conversion efficiencies of 9.6% for an eight-pair single-stage HH module and 12.4% for a HH/Bi_2_Te_3_ segmented module under temperature differences of 666 and 698 K, respectively. This work demonstrated that scalable material synthesis and module-topology optimization must be developed concurrently because nonuniform bulk properties, inappropriate leg geometry, interfacial resistance, and parasitic thermal losses can prevent a high material zT from being retained at the module level.

Yu et al. further combined p-type (Nb_0.8_Ta_0.2_)_0.8_Ti_0.2_FeSb with n-type Hf_0.5_Zr_0.5_NiSn_0.98_Sb_0.02_ in an eight-pair single-stage module designed using a full-parameter three-dimensional model.^203^ A conversion efficiency of 8.3% and a power density of 2.11 W cm^−2^ were obtained at hot- and cold-side temperatures of 997 and 342 K, respectively. The matched thermoelectric properties and thermal-expansion coefficients of the p-type and n-type legs contributed to the mechanical and thermal compatibility of the module. Beyond planar module optimization, Li et al. developed a 72-couple conformal HH module that could be directly integrated onto a cylindrical heat pipe.^124^ The module generated 56.6 W and reached a device power density of 3.13 W cm^−2^ under a temperature difference of 570 K, demonstrating that conformal integration can reduce the thermal penalty associated with intermediate heat exchangers and improve utilization of the available heat-source temperature difference.

High-temperature interface stability represents another decisive requirement for HH generators. Liu et al. used interfacial reaction energies and atomic migration barriers to screen Cr and Mo as thermally inert barrier materials for MNiSn- and MCoSb-based HH compounds.^204^ The resulting interfaces maintained specific contact resistivities below 1 μΩ cm^2^ after aging at 1,073 K for 30 days. Eight-pair single-stage HH and HH/Bi_2_Te_3_ segmented modules employing a Cr barrier achieved conversion efficiencies of 11.1% and 13.3% under temperature differences of 740 and 774 K, respectively. These studies indicate that the recent development of HH generators has progressed beyond isolated improvements in peak zT toward coordinated control of scalable material preparation, p-n compatibility, module geometry, heat-source conformity, and high-temperature ohmic contacts.

Skutterudites are commonly classified as intermediate-temperature thermoelectric materials; however, their useful operating range extends to approximately 873 K, and they frequently serve as the hot-side or intermediate-to-high-temperature segments of TEGs. Because the temperature boundaries adopted in this review are not absolute, skutterudites are discussed in the present high-temperature section according to their generator-level role near the medium- and high-temperature boundary. Filled CoSb_3_-based skutterudites benefit from guest-atom filling, carrier regulation, and multiscale phonon scattering, which can provide favorable average zT values over a broad temperature range. Their translation into generators, nevertheless, requires compatible p-type and n-type legs, stable segment-to-segment and leg-to-electrode interfaces, suppression of Sb loss, and reproducible module fabrication.

At the module level, Zhang et al. combined Bi_2_Te_3_-based alloys as the low-temperature segments with filled skutterudites as the high-temperature segments.^73^ Full-parameter three-dimensional optimization and low-loss interface integration enabled a conversion efficiency of 12% and a power density of 1.4 W cm^−2^ under a temperature difference of 541 K, corresponding to 96.9% of the theoretical efficiency predicted from the constituent materials. As shown in Figure 4G, Wan et al. constructed an all-skutterudite segmented module using compositionally related CoSb_3_-based materials for both the lower- and higher-temperature sections of the p-type and n-type legs.^200^ This same-parent design reduced mismatch in thermal-expansion coefficients and compatibility factors and enabled the segmented junctions to be fabricated by one-step sintering. The eight-pair module delivered a maximum output power of 4.3 W and a conversion efficiency of 10.4% at a hot-side temperature of 873 K and a temperature difference of 574 K. The segment interfaces retained negligible contact resistance after thermal aging at 723 K for 360 h, demonstrating that same-parent segmentation can broaden the effective operating range while avoiding some of the interfacial penalties associated with dissimilar thermoelectric materials.

Recent work has also extended skutterudite research from high-efficiency modules toward scalable manufacturing and application-specific integration. Shi et al. collected and consolidated all major products generated during melt spinning, increasing the usable material yield from approximately 55% to approximately 91%, while retaining homogeneous thermoelectric performance in large skutterudite bulks.^205^ A modified two-step sintering route produced barrier-layer/skutterudite joints with a contact resistivity of approximately 2 μΩ cm^2^ and a bonding strength of approximately 30 MPa, with no significant degradation after aging at 773 K for 30 days. The resulting eight-pair module achieved a conversion efficiency of 9% under a temperature difference of 580 K and maintained stable output during a 360-h test at a hot-side temperature of 773 K. Jing et al. further designed a high-aspect-ratio skutterudite module for a milliwatt radioisotope TEG.^206^ The fabricated 64-leg module produced an open-circuit voltage of 3.51 V, a maximum output power of 194.45 mW, and a conversion efficiency of 3.4% at hot- and cold-side temperatures of 723 and 296 K, respectively. Although the efficiency of this application-specific module was lower than that of optimized waste-heat modules, the study provided generator-level evidence concerning leg geometry, thermal resistance, environmental temperature, and thermomechanical stress. Collectively, these results show that skutterudite technology has advanced from material-level filling strategies to segmented-module design, interface aging, batch fabrication, and system-oriented demonstrations, while Sb volatilization, oxidation, long-term packaging stability, and application-scale thermal cycling remain important limitations.

Beyond HH alloys and skutterudites, Cu_2_X compounds, SiGe alloys, Zintl phases, and thermoelectric oxides provide complementary characteristics for high-temperature applications. Their relative value should be assessed using not only peak ZT but also oxidation resistance, elemental volatility, mechanical compatibility, raw material availability, and demonstrated module performance. As shown in Figure 4D, Weidi Liu et al.^197^ investigated Cu_2_X-based thermoelectric materials, systematically optimizing the carrier concentration via Cu vacancies, Br doping, and S doping, while incorporating secondary phases to suppress lattice thermal conductivity. The resulting Cu_2_X-based materials exhibited exceptionally high ZT values in the temperature range of 800–1,000 K. A thermoelectric module composed of Cu_2_Se and Yb_0_._3_Co_4_Sb_12_ was fabricated, achieving an energy conversion efficiency of approximately 9%. At 800 K, the module displayed a ZT of 2.4 and a power factor of 9.2 μW cm^−1^ K^−2^. Nanostructuring and the incorporation of carbon nanotubes further reduced the lattice thermal conductivity κ_l_ to 0.2 W m^−1^ K^−1^. In addition, Cu_2_S-based materials were evaluated due to the high abundance and low cost of sulfur, exhibiting a ZT of 1.7 at 1,000 K; the introduction of graphene further decreased the lattice thermal conductivity to 0.3 W m^−1^ K^−1^. Collectively, this work establishes a sound foundation for high-temperature thermoelectric materials and provides a pathway for the rational design and development of high-temperature TEGs. As shown in Figure 4E, Chenguang Fu et al.^198^ addressed the long-standing limitation of low ZT values in p-type HH alloys at high temperatures, particularly above 1,000 K, where their performance typically lags behind that of their n-type counterparts. The p-type FeNbSb system, despite its heavy-band structure favorable for high power factors, suffers from high lattice thermal conductivity, which constrains its ZT. In this work, Hf doping successfully increased the ZT to 1.5 at 1,200 K, setting a new record. The substantial mass and radius difference between Hf and Nb enhanced point-defect scattering, effectively lowering the lattice thermal conductivity. The resulting power factor ranged from 4.3–5.5 × 10^−3^ W m^−1^ K^−2^, and the lattice thermal conductivity was 1.0–1.5 W m^−1^ K^−1^ at 1,200 K. Using a module composed of eight n-p HH legs, a maximum power density of 2.2 W cm^−2^ was achieved. Overall, this study demonstrates that heavy-element doping can significantly improve the thermoelectric performance of HH alloys, offering practical insights for the development of high-temperature TEGs.

N. Mingo et al.^207^ investigated strategies to enhance the thermoelectric performance of SiGe alloys. By embedding 0.8% silicide nanoparticles into a Si_0_._5_Ge_0_._5_ alloy, the composite achieved a ZT of 1.7 at 900 K, indicating its potential for high-efficiency thermoelectric applications, including at room temperature. The SiGe + silicide system is compatible with complementary metal–oxide–semiconductor (CMOS) processing and can be monolithically integrated. Experimental results showed that at a nanoparticle volume fraction of 0.8%, the thermal conductivity of the composite decreased by 4–8 times relative to pure SiGe. An optimal nanoparticle size was also identified, which minimized thermal conductivity, with the ideal size ranging from a few nanometers to several tens of nanometers depending on the silicide type. This demonstrates the superiority of the alloy matrix over single crystals, as the broader distribution of phonon mean free paths allows nanoparticles to more effectively scatter phonons. This study provides both a material platform and a strategy for improving thermoelectric performance in high-temperature TEG applications.

Zintl phases and related rare-earth tellurides provide an important complementary route for very-high-temperature thermoelectric generation. In particular, p-type Yb_14_MnSb_11_ and n-type La_3−x_Te_4_ have been investigated as a potentially compatible material pair for operation over a broad temperature range extending toward approximately 1,273 K. These materials combine complex crystal structures, intrinsically strong phonon scattering, and composition-dependent carrier regulation. Their potential relevance to very-high-temperature generators arises not only from their peak zT values but also from their complementary conduction types and comparable thermal-expansion behavior. However, La_3−x_Te_4_ is more accurately described as a Th_3_P_4_-type rare-earth telluride rather than a conventional Zintl phase; it is discussed here together with Zintl compounds because of its close functional relationship with Yb_14_MnSb_11_ in high-temperature thermoelectric couples.

Yb_14_MnSb_11_ is a complex p-type Zintl phase with a tetragonal Ca_14_AlSb_11_-type structure. Its large unit cell and chemically complex anionic framework produce intrinsically low thermal conductivity, while its highly degenerate valence-band structure supports favorable high-temperature electronic transport. Yb_14_MnSb_11_-based materials have exhibited reported peak zT values of approximately 1.2–1.3 near 1,273 K. Grebenkemper et al. developed a scalable route based on ball milling, reaction with MnSb, annealing, and spark-plasma sintering, thereby avoiding the residual Sn and limited batch size associated with conventional flux growth.^208^ By introducing 5%–10% excess Mn, the carrier concentration was adjusted, and Yb_14_Mn_1.05_Sb_11_ achieved zT values of approximately 1.1–1.2 at 1,000 K. These results demonstrate that phase purity, Mn distribution, carrier concentration, and high-temperature compositional stability must be controlled simultaneously when translating Yb_14_MnSb_11_ into practical thermoelectric legs.

Rare-earth substitution provides an additional means of regulating both transport properties and environmental stability. Devlin et al. reported that Ce^3+^ substitution for Yb^2+^ reduced the hole concentration, increased the Seebeck coefficient, and reduced the thermal conductivity of Yb_14_MnSb_11_.^209^ The optimized Yb_13.6_Ce_0.4_MnSb_11_ composition reached a maximum zT of 1.21 at 1,200 K and an average zT of 0.80 over 475–1,275 K, compared with 0.71 for the unsubstituted material. Nevertheless, excessive carrier depletion promoted bipolar transport at the upper end of the temperature range. Lu substitution produced a similar trade-off: Yb_13.7_Lu_0.3_MnSb_11_ retained a peak zT of approximately 1.14 at 1,200 K, but further Lu addition reduced the peak performance and generated LuSb secondary phases when the solubility limit was exceeded.^210^ These results indicate that rare-earth substitution should be optimized using average zT, phase purity, and environmental stability rather than peak zT alone.

La_3−x_Te_4_ is a high-temperature n-type rare-earth telluride in which La vacancies provide intrinsic control of the electron concentration. The vacancy-rich Th_3_P_4_-type structure introduces strong phonon scattering, resulting in lattice thermal conductivities of approximately 0.4–0.8 W m^−1^ K^−1^. May et al. used mechanical alloying to produce compositionally homogeneous La_3−x_Te_4_ and obtained a maximum zT of approximately 1.13 at 1,275 K, exceeding that of the n-type SiGe materials used as the high-temperature benchmark at the time.^211^ The calculated thermoelectric compatibility factor of La_3−x_Te_4_ was also more favorable and less temperature dependent than that of SiGe. For a hot-side temperature of 1,273 K and a temperature difference of 900 K, the predicted efficiency of a La_3−x_Te_4_-PbTe segmented leg reached approximately 14.3%, while single-material La_3−x_Te_4_ and SiGe legs reached approximately 11.5% and 11.2%, respectively. In addition, the thermal-expansion coefficient of La_3−x_Te_4_ was reported to be comparable to that of p-type Yb_14_MnSb_11_, supporting their potential use as a high-temperature p-n material pair.

These results indicate that Yb_14_MnSb_11_ and La_3−x_Te_4_ can provide material-level thermoelectric performance competitive with, and in selected cases superior to, conventional SiGe. However, they should be regarded as potential alternatives rather than established replacements. Current evidence remains concentrated at the material and thermoelectric-leg levels, whereas stable electrodes, diffusion barriers, hermetic packaging, oxidation resistance, thermal-cycling durability, and complete p-n module demonstrations remain insufficiently established. Beyond these refractory bulk materials, flexible and structurally adaptive high-temperature generators provide a different route for matching unconventional heat-source geometries.

As shown in Figure 4F, Zhaojun Liu et al.^199^ developed a novel flexible TEG for high-temperature energy harvesting, drawing inspiration from biological structures, including pine cones, succulents, and feathers. The device employed In_2_O_3_ and Ag as thermoelectric materials and was fabricated using screen-printing techniques. Under a temperature gradient of 1,000 K, a single TEG demonstrated an output voltage density of 286.1 mV cm^−2^ and a maximum power density of 66.5 mW m^−2^. After over 1,400 cycles, the internal resistance increased by only 3.1%, indicating excellent mechanical flexibility and operational reliability. This bioinspired design provides a viable approach for flexible TEGs to achieve efficient energy harvesting in high-temperature environments.

Micro-combustion-driven TEGs represent a system-level route for converting the chemical energy of portable fuels into electricity through an intermediate high-temperature heat source.^212^^,^^213^^,^^214^^,^^215^ Hydrocarbon- or hydrogen-fueled systems have achieved reported electrical efficiencies of up to 3.78% and maximum output powers of 76.9 W. Biomass-fueled systems generally exhibit lower electrical efficiencies, but multi-module configurations have produced total output powers of up to 252 W. Propane-fueled systems have also demonstrated gravimetric energy densities higher than those of some battery systems, while the rejected heat can be used for combined heat and power operation.

These systems are attractive for remote power supply, emergency communication, outdoor equipment, and other applications in which rapid fuel replenishment and simultaneous heat production are advantageous. They may also provide distributed power for microelectromechanical systems and serve as a heat-source platform for evaluating thermoelectric modules under controlled high-temperature conditions. Their practical value, therefore, depends on the combined performance of the combustor, heat exchanger, thermoelectric module, cooling unit, and auxiliary components.

Important limitations remain at the system level. Incomplete combustion, nonuniform hot-side temperature distributions, limited heat-capture efficiency, noise, and pollutant emissions can reduce the overall benefit of micro-combustion systems. Commercial Bi_2_Te_3_-based modules cannot tolerate the highest combustor temperatures, whereas advanced high-temperature modules remain expensive and insufficiently commercialized. In addition, many reported prototypes rely on external air compressors or open cooling systems, which increase system complexity and auxiliary power consumption. Future development should, therefore, focus on self-sustained combustion, compact heat exchangers, thermally matched high-temperature modules, and closed-loop cooling.

Among the high-temperature material families considered in this review, HH alloys currently provide comparatively strong evidence across the material, single-leg, contact, and module levels. Band engineering and alloying have produced high power factors, while direct bonding, diffusion barriers, and module optimization have demonstrated that these materials can be translated into high power density and meaningful conversion efficiency. Skutterudites have also reached a comparatively advanced module stage, particularly in segmented architectures, although Sb volatilization, oxidation, and long-term interface stability remain important limitations. SiGe alloys retain strategic value for prolonged very-high-temperature operation because of their established thermal stability, but Ge cost and relatively high thermal conductivity constrain widespread terrestrial applications. Cu_2_X compounds can achieve high material-level ZT, whereas Cu-ion migration, compositional redistribution, and oxidation introduce substantial reliability uncertainty. Zintl phases and related rare-earth tellurides now provide a more clearly defined very-high-temperature material route than a generic emerging-material category. The combination of p-type Yb_14_MnSb_11_ and n-type La_3−x_Te_4_ offers material-level performance competitive with or superior to SiGe and favorable thermal-expansion compatibility. However, oxidation, elemental redistribution, stable joining, hermetic packaging, and long-duration p-n couple or module validation remain insufficient. These materials should, therefore, be regarded as potential SiGe alternatives for selected extreme-temperature applications rather than as established replacements. High-temperature flexible generators are also strategically promising, but their evidence remains concentrated at the prototype level. Representative high-temperature thermoelectric materials, fabrication processes, and performance metrics are summarized in Table 3.

**Table 3:** High-temperature thermoelectric generators

Thermoelectric material (n/p)	Fabrication process	ZT value	Power density	Power factor	Operating Temperature Range
TaFeSb-based half-Heusler compound^201^	Ball milling and hot pressing	1.52 (973 K)	–	52 μWcm^−1^K^−2^ (300–973 K)	300–973 K
Nb_1−x_Ti_x_FeSb^202^	Arc melting; ball milling; hot pressing	1.1 (973 K)	22 Wcm^−2^ (ΔT = 575 K)	106 μWcm^−1^K^−2^ (300 K)	293–973 K
Si_0_._5_Ge_0_._5_ alloy matrix + nanoparticles^207^	–	1.7 (900 K)	–	–	300–900 K
Fe_1_._05_Nb_0_._75_Ti_0_._25_Sb^216^	Arc melting; ball milling; hot pressing	1.34 (1,150 K)	–	3.0 mWm^−1^K^−2^ (1,150 K)	300–1,550 K
Hf_0_._8_Ti_0_._2_CoSb_0_._8_Sn_0_._2_^217^	Arc melting → ball milling (nanostructuring) → DC hot pressing	1.0 (1,073 K)	–	25.7 μWcm^−1^K^−2^ (1,073 K)	300–1,073 K
(Hf_0_._6_Zr_0_._4_)NiSn_0_._99_Sb_0_._01_ + 5wt%W^218^	Radio-frequency (RF) induction melting + ball milling + SPS	1.4 (873 K)	13.93 Wcm^−2^ (ΔT = 674 K)	62.3 μWcm^−1^K^−2^ (773 K)	300–973 K
Nb_0_._86_Hf_0_._14_FeSb^219^	Induction melting + ball milling + hot pressing	1.2 (1,050 K)	3.1Wcm^−2^ (ΔT = 680 K)	45 μWcm^−1^K^−2^ (1,050 K)	300–1,050 K
Zr_0_._6_(NbTa)_0_._4_CoSb^220^	Levitation melting + ball milling + SPS	0.42 (923 K)	–	1.5 × 10^−3^ Wcm^−1^K^−2^ (923 K)	323–923 K
TiCo_0_._85_Fe_0_._15_Sb_0_._97_Sn_0_._03_^221^	Arc melting + ball milling + SPS	0.4 (873 K)	–	1.94 mWm^−1^K^−2^ (825 K)	300–873 K
(Nb_0_._64_Ta_0_._36_)_0_._8_Ti_0_._2_FeSb^222^	Magnetic levitation melting + SPS	1.6 (1,200 K)	–	–	300–1,200 K

### Transferability of design strategies across temperature regimes

Design strategies developed in one temperature regime can provide useful principles for another regime, although the specific materials and processing routes cannot always be transferred directly. Low-temperature flexible generators demonstrate the importance of compliant interconnects, strain release, conformal heat-source contact, and low-cost printing. These principles are relevant to high-temperature generators exposed to curved surfaces or repeated thermal strain. However, polymer substrates, organic encapsulants, and low-melting-point conductors used in wearable devices must be replaced by thermally stable metallic or ceramic components.

Medium-temperature studies provide particularly relevant strategies for high-temperature development. Band engineering, carrier-concentration regulation, segmented architectures, metallization, and diffusion barriers can all be extended to higher operating temperatures. Their implementation must, nevertheless, account for stronger elemental diffusion, intrinsic carrier excitation, oxidation, and thermal-expansion mismatch. A barrier material that is stable in a medium-temperature module may react rapidly or lose mechanical integrity at higher temperatures.

Nanostructuring and multiscale phonon scattering are common material strategies across all temperature regimes. At low and medium temperatures, nanoscale interfaces can effectively reduce lattice thermal conductivity. At high temperatures, however, grain growth, precipitate coarsening, and defect redistribution may weaken the phonon-scattering effect during prolonged operation. The transferability of a strategy should, therefore, be evaluated according to both its initial performance benefit and its structural stability within the intended operating environment.

Overall, the most transferable concepts are the coordinated control of carrier transport, phonon transport, interfacial resistance, and thermal stress. The least transferable elements are temperature-sensitive substrates, binders, solders, encapsulants, and metastable nanostructures. Cross-temperature design should, therefore, focus on transferring physical principles rather than directly reproducing specific material combinations or fabrication processes.

### Application requirements and material-to-system translation

Material-level thermoelectric performance is converted into usable electrical output through a sequence of coupled processes: heat capture from the source, heat transfer through the hot-side heat exchanger and interfaces, thermoelectric conversion within the p-type and n-type legs, electrical transport through the contacts and interconnects, cold-side heat rejection, and power conditioning. Losses at any of these stages can reduce the usable system output even when the thermoelectric materials exhibit a high peak zT. Consequently, the practical relevance of a material should be assessed using its average properties over the operating range together with p-n compatibility, contact resistance, module geometry, effective temperature difference, cooling requirements, and auxiliary power consumption.

Low-temperature thermoelectric applications include body-heat harvesting for wearable electronics, self-powered physiological and environmental sensors, Internet-of-Things nodes, cold-chain monitoring, and low-grade heat recovery from electronic equipment.^24^^,^^30^^,^^31^^,^^32^^,^^33^^,^^74^^,^^75^^,^^76^^,^^77^^,^^78^^,^^79^^,^^80^^,^^81^^,^^82^^,^^83^^,^^84^^,^^85^^,^^86^^,^^87^^,^^88^^,^^89^^,^^90^^,^^91^^,^^92^^,^^93^^,^^94^^,^^95^^,^^96^^,^^97^^,^^98^^,^^151^^,^^152^^,^^153^^,^^154^^,^^155^^,^^156^^,^^157^^,^^158^^,^^159^^,^^160^^,^^161^ These applications generally operate under small source-to-ambient temperature differences, and the temperature drop established across the thermoelectric legs can be substantially smaller than the nominal temperature difference. Their performance, therefore, depends strongly on conformal heat-source contact, low thermal and electrical contact resistance, suppression of parasitic heat leakage, mechanical compliance, and ultra-low-power energy-management circuits. Bi_2_Te_3_- and Ag_2_Se-based materials and small rigid or flexible devices have reached a comparatively advanced level of development. However, broader deployment of wearable generators still requires reproducible output under realistic on-body conditions; long-term resistance to bending, perspiration, humidity, and encapsulation failure; scalable device fabrication; and integration with energy storage and power-management electronics.

Medium-temperature applications primarily include automotive exhaust recovery, boilers, industrial flue gases, steam and process pipelines, and waste-heat recovery in the steel, chemical, cement, and manufacturing industries.^33^^,^^165^^,^^166^^,^^167^^,^^168^^,^^169^^,^^170^^,^^171^^,^^172^^,^^173^^,^^174^^,^^175^^,^^176^^,^^177^^,^^178^^,^^179^^,^^181^^,^^183^^,^^184^^,^^185^^,^^186^^,^^187^ Compared with low-temperature systems, the larger available temperature difference enables higher electrical output, but it also introduces vibration, repeated thermal cycling, interfacial diffusion, and more demanding heat-exchanger requirements. PbTe-, PbSe-, GeTe-, SnTe-, and Mg_3_Sb_2_-based materials have achieved high material-level performance, while diffusion barriers and segmented modules have demonstrated that these improvements can be transferred to generator efficiency. The principal barriers to higher maturity are long-term contact stability, the environmental and supply concerns associated with Pb- and Te-containing materials, reproducible large-scale densification, manufacturing yield, module cost, and maintenance of the effective temperature difference under realistic heat-source conditions. Medium-temperature technology can, therefore, be regarded as being at the module-demonstration and early application-integration stage rather than at the level of widespread industrial deployment.

High-temperature applications include industrial furnaces, rotary kilns, concentrated-solar receivers, gas-turbine and high-temperature exhaust systems, aerospace and radioisotope power sources, and micro-combustion-based remote power generators.^58^^,^^72^^,^^73^^,^^124^^,^^182^^,^^189^^,^^190^^,^^191^^,^^192^^,^^193^^,^^194^^,^^195^^,^^196^^,^^197^^,^^198^^,^^199^^,^^201^^,^^202^^,^^207^^,^^212^^,^^213^^,^^214^^,^^215^^,^^216^^,^^217^^,^^218^^,^^219^^,^^220^^,^^221^^,^^222^^,^^223^^,^^224^^,^^225^^,^^226^^,^^227^^,^^228^^,^^229^^,^^230^^,^^231^^,^^232^^,^^233^^,^^234^^,^^235^^,^^236^^,^^237^^,^^238^ These applications provide high-energy-density heat sources but impose simultaneous thermal-, chemical-, mechanical-, and system-level constraints. HH alloys and skutterudites currently provide comparatively strong module-level evidence, while SiGe retains an established role in selected space-power applications. Cu_2_X compounds, Zintl phases, related rare-earth tellurides, and thermoelectric oxides provide additional material options but generally have less complete module-level validation. Further maturation requires oxidation and volatilization control, chemically stable low-resistance contacts, reliable high-temperature joining and hermetic packaging, thermal-shock and vibration resistance, scalable module assembly, and heat collectors and cooling systems adapted to the target heat source. Long-duration operation under realistic atmospheres remains more important than further isolated increases in peak zT.

Technological maturity should, therefore, be assessed using an evidence chain rather than material zT alone. The first level is reproducible material performance at a relevant component size and over the full operating temperature range. The second is compatibility between p-type and n-type legs together with stable electrical and thermal interfaces. The third is complete-module evidence, including conversion efficiency, output power, power density, contact resistance, and effective temperature difference under specified boundary conditions. The fourth is reliability evidence obtained through isothermal aging, thermal cycling, mechanical loading, and atmosphere-dependent testing. The final level is application integration, including heat-source coupling, cooling, auxiliary power consumption, manufacturing yield, cost, maintenance, and system-level net output.

Under this framework, low-temperature thermoelectric materials and small devices are comparatively mature, although practical power output and wearable durability remain limiting. Medium-temperature technologies have progressed to successful module demonstrations, but cost, cycling reliability, and industrial heat-source integration still restrict widespread deployment. High-temperature technologies remain at an earlier engineering stage because material performance, environmental stability, joining, packaging, manufacturing, and thermal management must be satisfied simultaneously. These maturity differences also explain why the following section, “challenges of high-temperature TEGs,” focuses on the coupled material-, interface-, module-, and system-level barriers that currently limit high-temperature TEGs.

## Challenges of high-temperature TEGs

High-temperature thermoelectric materials and generators are attractive for recovering industrial waste heat and supplying power in extreme environments. However, increasing the operating temperature changes the dominant limitations of thermoelectric conversion. In addition to the coupled electrical and thermal transport properties of the thermoelectric materials, high-temperature operation introduces chemical instability, interfacial reactions, thermomechanical stress, demanding thermal-management requirements, and manufacturing difficulties. These factors create substantial differences between the performance measured for a thermoelectric material and that achieved by a complete generator. The main challenges are, therefore, discussed below at the material, interface, module, system, and manufacturing levels.^223^^,^^224^^,^^225^^,^^226^^,^^227^^,^^228^

### Material performance and long-term stability

The first challenge is to maintain favorable thermoelectric transport properties over a broad high-temperature range. Intrinsic carrier excitation and bipolar transport become increasingly important as the temperature rises and can reduce the Seebeck coefficient while increasing the electronic contribution to thermal conductivity. Meanwhile, electrical conductivity, the Seebeck coefficient, and thermal conductivity remain strongly coupled, making independent optimization difficult. A high peak ZT measured within a narrow temperature interval, therefore, does not necessarily indicate high generator efficiency. For practical high-temperature power generation, the average ZT over the entire operating range is generally more relevant.

The microstructures used to improve thermoelectric performance must also remain stable during prolonged thermal exposure. Nanoprecipitates, dense dislocation structures, grain boundaries, and metastable phases can effectively scatter phonons and reduce lattice thermal conductivity.^239^^,^^240^^,^^241^^,^^242^^,^^243^^,^^244^^,^^245^^,^^246^^,^^247^ At elevated temperatures, however, these structures may undergo coarsening, grain growth, phase transformation, or defect redistribution. Such changes can increase thermal conductivity or alter the carrier concentration, causing the thermoelectric properties to degrade with service time. Consequently, short-duration measurements during a single heating cycle are insufficient for assessing high-temperature applicability.

Chemical stability represents another fundamental limitation. Materials containing volatile elements such as Te, Se, Sb, or Cu may experience compositional changes during prolonged operation. Element loss can modify the carrier concentration, induce secondary phases, and reduce electrical performance. Exposure to oxygen or corrosive gases may additionally generate electrically resistive surface layers and alter the contact between the thermoelectric legs and electrodes. High-temperature material evaluation should, therefore, include isothermal aging, repeated thermal cycling, phase and microstructure analysis, mass-change measurements, and thermoelectric-property retention after aging.

### Material-family-specific bottlenecks

Different high-temperature thermoelectric material families exhibit distinct advantages and limitations. HH alloys generally possess high power factors, favorable mechanical properties, and comparatively good thermal stability. Their major limitations include relatively high lattice thermal conductivity, the cost of compositions containing Hf or Ta, oxidation under prolonged exposure, and difficulties in forming stable low-resistance contacts. Although alloying and nanostructuring have substantially improved their material-level ZT, module performance remains sensitive to metallization, diffusion barriers, and thermomechanical compatibility.^226^^,^^240^^,^^241^^,^^242^^,^^243^^,^^244^^,^^245^^,^^248^^,^^249^^,^^250^^,^^251^^,^^252^^,^^253^^,^^254^^,^^255^^,^^256^^,^^257^^,^^258^^,^^259^^,^^260^^,^^261^^,^^262^^,^^263^^,^^264^^,^^265^^,^^266^^,^^267^^,^^268^^,^^269^

Skutterudites provide a broad operating range and can achieve reduced lattice thermal conductivity through filling and multiscale phonon scattering. Their practical development is constrained by Sb volatilization, oxidation, the performance difference between p-type and n-type compositions, and the stability of the interfaces between skutterudite legs and metallic electrodes. Filled skutterudites have demonstrated considerable potential in segmented modules, but long-term chemical stability and scalable packaging remain important requirements.^224^^,^^270^^,^^271^

SiGe alloys have a comparatively long history in high-temperature and space applications and exhibit good compatibility with semiconductor processing.^246^^,^^247^ Their limitations include the cost of Ge, relatively high thermal conductivity, compositional segregation during processing, and the complexity of nanostructure fabrication. Zintl phases and related rare-earth tellurides, particularly p-type Yb_14_MnSb_11_ and n-type La_3−x_Te_4_, provide a complementary material pair for very-high-temperature power generation. Their material-level performance can equal or exceed conventional SiGe over selected temperature ranges, but oxidation, Sb or Te redistribution, mechanical brittleness, contact formation, hermetic packaging, and limited long-duration module validation remain critical constraints. Cu_2_X compounds and thermoelectric oxides offer complementary characteristics. Cu_2_X materials can exhibit high ZT, but Cu-ion migration, elemental redistribution, and oxidation can degrade device stability. Oxide thermoelectrics have advantages in oxidation resistance, elemental abundance, and high-temperature chemical stability, although their electrical conductivity and module efficiency are generally lower than those of leading intermetallic and chalcogenide systems. Material selection should, therefore, consider operating atmosphere, required lifetime, raw-material availability, and module compatibility in addition to peak ZT. The principal characteristics, performance advantages, limitations, costs, and applicable temperature ranges of representative high-temperature thermoelectric material families are compared in Table 4.

**Table 4:** Commonly used high-temperature thermoelectric materials^271^

Material	Material type	ZT value	Synthesis method	Performance advantages	Performance challenges	Cost	Applicable temperature range	Applications
Skutterudite compounds	AB_3_-type, e.g., CoSb_3_	1.45	Mechanical alloying; ball milling-hot pressing	High Seebeck coefficient; good electrical transport properties	Sb volatilization and oxidation; p-n performance mismatch; interface and packaging stability.	Moderate, due to the presence of relatively rare elements	500–900 K	Industrial waste-heat recovery; automotive exhaust waste-heat utilization
Half-Heusler compounds	XYZ-type, e.g., TiNiSn	1.0	Mechanical alloying; arc melting-hot pressing	Good high-temperature stability; suitable for high-temperature applications	High thermal conductivity; further reduction is required to improve ZT	Moderate; constituent materials are relatively inexpensive	473–1,073 K	Industrial waste-heat recovery; high-temperature sensors
Nanostructured oxides	e.g., NaCo_2_O_4_	0.65	Sol-gel processing; electrospinning-SPS	High thermal stability; good oxidation resistance	Relatively low electrical conductivity, requiring further optimization	Low, owing to the use of common metal oxides	300–1,000 K	High-temperature sensors; solar thermoelectric energy conversion
Si-Ge binary systems	SiGe alloys	1.3	Ball milling-hot pressing; mechanical alloying-hot pressing	Good high-temperature stability; suitable for high-temperature applications	High thermal conductivity; nanostructuring processes are complex	Moderate, due to the high cost of germanium	300–1,200 K	Industrial waste-heat recovery; thermoelectric generators for space exploration

### Oxidation, volatilization, and environmental stability

Environmental stability should be distinguished from intrinsic thermal stability. A thermoelectric material may retain its crystal structure and transport properties during heating in vacuum or an inert atmosphere but degrade rapidly in air because of oxidation, volatile-element loss, or reactions between the oxide layer and the underlying matrix. Oxidation behavior is governed not only by the nominal operating temperature but also by the oxygen partial pressure, exposure time, temperature gradient, surface condition, and the mechanical integrity of the reaction layer. Accordingly, high-temperature stability should be evaluated using mass-change kinetics, oxide-layer composition and thickness, microcrack formation, post-exposure transport properties, and device-level output retention.

HH alloys exhibit strongly composition-dependent oxidation behavior. Kang et al. compared n-type (Hf_0.6_Zr_0.4_)NiSn_0.99_Sb_0.01_, p-type (Hf_0.4_Zr_0.4_Ti_0.2_)CoSb_0.8_Sn_0.2_, and p-type (Nb_0.6_Ti_0.4_)FeSb_0.95_Sn_0.05_ after exposure to air at 873 K.^259^ After 72 h, the MNiSn and NbFeSb samples developed oxide layers of approximately 20–30 and 5 μm, respectively, whereas the oxidized region of MCoSb extended to approximately 268 μm. The comparatively high stability of MNiSn was attributed to an underlying Ni-Sn intermetallic layer, while NbFeSb formed a Nb-rich sublayer beneath an external TiO_2_ layer that restricted oxygen migration. In contrast, the continuous oxidation of MCoSb increased its electrical resistivity by more than 250%. This difference was retained at the device level: an MNiSn/MCoSb unicouple lost approximately 50% of its output power after 5 days of operation in air at a hot-side temperature of 823 K, whereas an MNiSn/NbFeSb unicouple exhibited only approximately 7% degradation after 7 days at 873 K.

Oxidation resistance can also vary non-monotonically along the temperature gradient of a thermoelectric leg. Wang et al. found that Zr_0.5_Hf_0.5_NiSn_0.985_Sb_0.015_ underwent severe oxidation at intermediate temperatures of 723–773 K, although its oxidation rate was lower at 823–1,073 K.^272^ At 723–773 K, competitive inward oxygen diffusion and outward elemental transport produced alternating (ZrO_2_ + HfO_2_ + SnO_2_)/Ni_3_Sn_2_ layers containing abundant pores and cracks. At higher temperatures, a comparatively dense layer composed mainly of ZrO_2_, HfO_2_, and Ni_3_Sn_2_ suppressed further oxygen penetration. Pre-oxidation at 873 K under flowing Ar was, therefore, used to form an approximately 24-μm artificial dense oxide layer. An untreated single-leg device retained only 61% of its initial maximum output power after 15 days in air at a temperature gradient of 778/300 K, whereas the pre-oxidized device exhibited negligible output degradation. These results indicate that oxidation assessment at a single temperature may overlook the most vulnerable region of an operating thermoelectric leg and that controlled pre-oxidation can convert selected reaction products into a protective surface layer.

The environmental degradation of skutterudites involves coupled oxidation, Sb transport, and volatilization. For unfilled CoSb_3_, oxide growth between 500°C and 600 °C followed approximately parabolic kinetics, and the surface layer consisted mainly of Sb_2_O_3_ and CoSb_2_O_6_.^273^ At 650 °C, sublimation of Sb_2_O_3_ caused substantial mass loss and produced a two-layer scale comprising an Sb_2_O_3_-rich outer layer and an inner Sb_2_O_3_/CoSb_2_O_6_ mixture. Thermal-expansion mismatch generated cracks and local detachment at the scale/CoSb_3_ interface, providing rapid pathways for continued oxidation. After 48 h at 650 °C, the room temperature electrical conductivity decreased by approximately 24%, and the zT value at 327 °C decreased from 0.24 to 0.17. This result demonstrates that the degradation of CoSb_3_ is controlled by both oxide formation and the loss of Sb through volatile oxidation products.

Filling the skutterudite cages does not necessarily improve environmental stability. Xia et al. showed that Yb_y_Co_4_Sb_12_ begins to oxidize significantly above approximately 650 K.^274^ Its oxidation proceeds through a repetitive sequence of dense oxide-layer formation, thermal-stress release, microcrack generation, accelerated transport of oxygen and Sb, partial crack filling, and renewed oxide-layer growth. Increasing the Yb filling fraction monotonically reduced the oxidation activation energy because of the high oxygen affinity of Yb and the relatively weak bonding between Yb and the Co_4_Sb_12_ framework. Thus, the filler atoms that reduce lattice thermal conductivity can simultaneously reduce long-term stability in air. The p-type skutterudite CeFe_4_Sb_12_ exhibited an even greater susceptibility to oxidation.^275^ A measurable mass gain was observed at 600 K, and an oxide layer approximately 200 μm thick formed after only 1 h at 900 K. The rapid degradation was attributed to preferential oxidation of Ce and oxygen-induced decomposition of the unstable “FeSb_3_” framework into FeSb_2_ and Sb. These findings indicate that p-type and n-type skutterudite legs may exhibit substantially different environmental lifetimes even when their thermoelectric properties are compatible.

Protective coatings can suppress oxygen ingress or volatile-element loss, but their chemical and thermomechanical compatibility must also be considered. Dong et al. fabricated glass-particle- and Al_2_O_3_-particle-dispersed hybrid silica coatings on n-type Yb_0.3_Co_4_Sb_12_ and p-type CeFe_3_CoSb_12_.^276^ Particle addition reduced shrinkage stress and enabled the formation of relatively thick, dense coatings. However, the glass-containing coating underwent Sn diffusion toward the skutterudite and Sb diffusion toward the coating during aging at 873 K, eventually forming Sn-Sb-containing reaction products and losing its protective function. In contrast, the Al_2_O_3_-silica coating exhibited no detectable interdiffusion or reaction after aging at 873 K for 62 h and effectively suppressed Sb sublimation. Because these aging experiments were performed in vacuum, the results directly demonstrate control of Sb loss and coating-matrix compatibility rather than complete oxidation protection in air. Atmosphere-specific validation is, therefore, required before a coating can be considered suitable for practical generator operation.

Zintl phases based on Yb_14_MSb_11_ also exhibit a pronounced difference between intrinsic high-temperature stability and stability in oxygen-containing environments. Justl and Kauzlarich compared Yb_14_MSb_11_ with M = Mg, Mn, and Zn during heating from room temperature to 1,000 °C in dry air.^277^ Oxidation initially proceeded slowly but accelerated markedly near 400°C. After high-temperature exposure, the pellets developed a layered degradation structure consisting of a Yb_2_O_3_-rich outer scale, a porous inner region enriched in Sb-containing phases, and a partially retained Yb_14_MSb_11_ core. Inward oxygen diffusion and outward transport of Sb or Sb-oxide-containing species caused delamination and pore formation. At approximately 785°C–803 °C, melting and solidification associated with YbSb_2_ were observed, and molten reaction products were extruded from the Yb_14_MgSb_11_ and Yb_14_MnSb_11_ samples. Yb_14_ZnSb_11_ showed the smallest overall oxidation response, but none of the three compositions remained chemically intact after prolonged exposure to air at 1,000 °C.

Rare-earth substitution may alter the oxidation kinetics, but the resulting protection is strongly temperature and cycle dependent. In a separate thermogravimetric protocol, both Yb_14_MnSb_11_ and Yb_13.6_Ce_0.4_MnSb_11_ showed no pronounced oxidation signal until approximately 700 K, and the Ce-substituted sample exhibited no detectable degradation after 77 days in ambient air at room temperature.^209^ The more rapid formation of a rare-earth-containing oxide scale may improve passivation, although this conclusion cannot be extrapolated directly to prolonged operation at the highest temperatures. Lu substitution provides a similar example of this trade-off. After four oxidation cycles to 400 °C in dry air, Yb_13.7_Lu_0.3_MnSb_11_ retained approximately 96 wt. % of the main phase, compared with approximately 81 wt. % for unsubstituted Yb_14_MnSb_11_, suggesting partial protection by a Lu-containing oxide shell.^210^ However, repeated cycling produced renewed exothermic behavior, indicating that the initially passivating film may crack, while high-temperature oxidation still generated YbSb_2_, YbMnSb_2_, and molten reaction products. Rare-earth substitution, therefore, introduces a coupled trade-off among carrier concentration, peak zT, oxide-layer formation, and long-term mechanical integrity.

La_3−x_Te_4_ is even more sensitive to residual oxygen despite its favorable intrinsic thermal stability. Li et al. found that La_2.74_Te_4_ remained comparatively stable up to approximately 1,200 K under high-purity Ar but began to oxidize above approximately 700 K in air or under an oxygen partial pressure as low as 10^–3^ MPa.^278^ In air, the sample mass increased sharply to approximately 122% of its initial value near 850 K. The resulting La-O-Te surface layer contained cracks and was susceptible to spallation, repeatedly exposing fresh material to oxygen. Incorporating metallic Ni particles substantially reduced the oxidation rate. For La_2.74_Te_4_/Ni composites, measurable oxidation under P_O2_ = 10^–3^ MPa was delayed to approximately 900 K, and the total mass gain remained below approximately 2%. The improvement was attributed to a dense and adherent composite scale containing La_2_NiO_4_, Ni_2_Te_3_O_8_, and NiO, together with an underlying La_4_Te_7_-rich layer. The 10 vol. % Ni composite retained a zT of approximately 1.0 at 1,273 K, indicating that internal composite design can enhance oxidation resistance without eliminating useful high-temperature thermoelectric performance. Nevertheless, the added metallic phase, oxide-layer evolution, and long-term electrical-contact compatibility require further module-level validation.

Collectively, these studies show that no universal oxidation ranking can be assigned to an entire thermoelectric material family. HH stability depends strongly on composition, oxidation-layer architecture, and position within the operating temperature gradient. Skutterudite degradation is coupled to filler chemistry, Sb transport, volatile oxide formation, and scale cracking. Yb_14_MSb_11_-based Zintl phases can form partially passivating rare-earth oxide scales, but continued Sb redistribution, phase decomposition, and oxide-layer fracture remain important failure mechanisms. La_3−x_Te_4_ combines favorable intrinsic high-temperature transport with extreme sensitivity to residual oxygen, although Ni-assisted formation of a dense composite oxide layer provides a promising protection strategy. Oxidation activation energies, onset temperatures, and mass changes reported using different sample forms, temperature profiles, atmospheres, and kinetic methods should, therefore, not be compared directly. Future studies should report oxygen partial pressure, temperature gradients, exposure time, scale composition and thickness, post-exposure transport properties, contact compatibility, and device-output retention. Protective strategies should additionally be evaluated under repeated thermal cycling and realistic module atmospheres rather than only through short-term material-level tests.

### Module efficiency, interfacial degradation, and thermomechanical reliability

Module conversion efficiency is governed by the integrated performance of the complete p-n assembly rather than by the peak ZT of an individual material. The average thermoelectric properties over the operating temperature range, compatibility between p-type and n-type legs, leg geometry and fill factor, ECR and TCR, parasitic heat leakage, and electrical load matching jointly determine the measured efficiency. Increasing the number or packing density of thermoelectric legs can increase the output voltage and heat-transfer area but may also increase internal resistance, thermal bypass, and thermomechanical stress. Meaningful comparisons of module efficiency should, therefore, specify the hot- and cold-side temperatures, effective temperature difference, module geometry, input-heat measurement method, electrical load condition, and auxiliary cooling consumption.

High material performance does not ensure high module efficiency because every thermoelectric leg must be connected to electrodes through metallization layers, diffusion barriers, solders, or brazing materials. At high temperatures, elemental interdiffusion and metallurgical reactions can form secondary phases with high electrical or thermal resistance. The resulting increase in contact resistance reduces the output power and can gradually offset improvements achieved at the material level.

A suitable diffusion barrier must satisfy several requirements simultaneously. It should suppress elemental transport, exhibit low ECR and TCR, maintain chemical compatibility with the thermoelectric material and electrode, and retain sufficient bonding strength during prolonged operation. Barrier-layer performance should, therefore, be evaluated after high-temperature aging rather than only in the as-fabricated state. Measurements of specific contact resistivity, reaction-layer thickness, bonding strength, and electrical-property retention are particularly important.

Thermal-expansion mismatch creates an additional reliability problem. Thermoelectric legs, metallic electrodes, ceramic substrates, barrier layers, and solders generally possess different coefficients of thermal expansion. Repeated heating and cooling produce cyclic shear and normal stresses at their interfaces, which can cause cracking, delamination, solder fatigue, or loss of electrical continuity. Potential solutions include compliant metallic interconnects, graded barrier layers, reduced-stiffness substrates, optimized leg geometry, and materials with better-matched thermal-expansion coefficients. Thermomechanical simulation should be combined with thermal-cycling experiments to identify the locations and mechanisms of failure.^229^^,^^259^^,^^260^^,^^261^^,^^262^^,^^263^^,^^270^^,^^279^^,^^280^^,^^281^^,^^282^

### Thermal management and application-specific constraints

The performance of a thermoelectric module depends on the temperature difference actually established across the thermoelectric legs rather than the nominal temperature of the heat source. Inadequate hot-side heat transfer, excessive cold-side thermal resistance, TCR, and parasitic heat leakage can substantially reduce the effective temperature difference. High-temperature system design must, therefore, coordinate the heat collector, heat exchanger, module thermal resistance, cooling structure, mechanical attachment, and operating environment.

Cold-side thermal management is particularly important. As the heat-source temperature and heat flux increase, passive cooling may become insufficient, whereas active cooling introduces auxiliary power consumption, additional components, and maintenance requirements. The cooling method should, therefore, be selected according to the expected heat flux, available installation space, ambient conditions, and allowable parasitic power. System efficiency should be calculated after accounting for the energy consumed by pumps, fans, or other auxiliary equipment.

Industrial rotary kilns illustrate the application-specific difficulties of high-temperature thermoelectric recovery.^230^^,^^231^^,^^232^^,^^233^^,^^234^ Direct attachment of rigid modules is restricted by continuous rotation, curved surfaces, vibration, dust, corrosive gases, and large temperature fluctuations. Poor conformity between a rigid module and a curved surface increases TCR, while repeated temperature changes accelerate interfacial fatigue. Noncontact radiative heat collection avoids direct attachment to the rotating shell but introduces an additional heat-transfer stage and may reduce the amount of heat delivered to the module. Practical kiln systems, therefore, require conformal or noncontact heat collectors, sealed module structures, mechanically compliant connections, and cooling systems that tolerate dust and limited maintenance.

Similar application-specific constraints arise in aerospace and concentrated-solar systems. Aerospace generators require low mass, resistance to vibration and radiation, and long service without maintenance. Concentrated-solar systems experience nonuniform heat flux and repeated thermal transients, requiring heat spreading and protection against local overheating. These examples demonstrate that the optimal thermoelectric material cannot be selected independently of the intended heat source and system architecture.

### Cost and scalable manufacturing

Cost and manufacturing scalability constitute challenges that are distinct from thermoelectric performance. Several high-temperature material systems contain expensive or supply-sensitive elements, including Hf, Ta, Ge, and Te. In addition, laboratory-scale preparation frequently relies on vacuum melting, repeated ball milling, hot pressing, spark-plasma sintering, or high-precision thin-film deposition. These methods provide effective control of composition and microstructure, but their batch nature, equipment requirements, and limited throughput complicate large-scale production.

Nanostructuring introduces an additional manufacturing trade-off. Nanoscale precipitates, grain refinement, and multiscale defects can reduce lattice thermal conductivity, but the corresponding powder synthesis, consolidation, and process control may increase cost and batch variability. Moreover, structures optimized in small specimens may be difficult to reproduce uniformly in large thermoelectric legs. The scalability of a material strategy should, therefore, be evaluated through production rate, material utilization, process yield, dimensional tolerance, batch-to-batch performance variation, and long-term microstructural stability.

Promising manufacturing routes include self-propagating synthesis, continuous powder processing, hot extrusion or rolling, near-net-shape forming, screen printing, additive manufacturing, automated leg cutting, and continuous joining. These techniques may reduce material waste and manual assembly, although high-temperature inks, binders, barrier layers, and joining materials require further development. Recycling cutting waste and rejected modules may additionally reduce dependence on expensive raw materials.

Module standardization is equally important. Customized module geometries improve adaptation to individual heat sources but increase design, tooling, and assembly costs. A practical manufacturing strategy should combine standardized thermoelectric submodules with application-specific heat collectors and cooling components. This approach can preserve heat-source adaptability while improving production volume, quality control, repairability, and replacement efficiency.^235^^,^^283^^,^^284^^,^^285^^,^^286^

Taken together, engineering-oriented thermoelectric studies should report a consistent set of module- and system-level metrics. These include conversion efficiency under specified hot- and cold-side temperatures, output power and power density, specific ECR and TCR, effective module temperature difference, thermal-cycling or isothermal-aging duration, post-aging performance retention, manufacturing yield, material utilization, and batch-to-batch variation. Reporting these parameters together would allow material improvements to be distinguished from gains resulting from module geometry, thermal boundary conditions, or test methodology.

### Research priorities, bottlenecks, and transformative approaches

On the basis of the available material- and module-level evidence, four bottlenecks should receive the highest priority: broad-temperature-range performance retention, chemical and oxidation stability, low-resistance and thermomechanically compatible interfaces, and scalable module fabrication. These bottlenecks are more immediate than the pursuit of isolated peak zT records because they directly determine whether high-temperature materials can operate reliably in complete generators.

In the near term, high-temperature thermoelectric research should prioritize interface stability and reproducible reliability evaluation. Diffusion-barrier selection should combine thermodynamic prediction with experimental measurements of contact resistivity, reaction-layer growth, bonding strength, and thermal-cycling lifetime. Standardized aging procedures are required so that stability results from different materials and modules can be meaningfully compared. Material studies should report average ZT, property retention, and atmosphere-dependent stability in addition to peak ZT.

In the medium term, material optimization should be integrated with scalable fabrication. Band engineering, high-entropy alloying, defect regulation, and hierarchical phonon scattering remain promising, but their value should be demonstrated after prolonged high-temperature exposure and at a relevant component size. Manufacturing research should focus on near-net-shape processing, continuous densification, automated joining, and improved batch consistency. Module development should simultaneously address p-n material matching, low-resistance contacts, thermomechanical compatibility, and packaging.

In the longer term, transformative approaches may extend the design space beyond incremental optimization of existing materials and modules. High-throughput computation, machine learning, and inverse design can accelerate the identification of compositions with high average ZT, reduced dependence on critical elements, and improved environmental stability. Self-protective surfaces, adaptive diffusion barriers, and self-healing interfaces may further mitigate oxidation and interfacial degradation during prolonged operation. Integrated co-design of thermoelectric modules, heat collectors, cooling systems, packaging, and power-management circuits may also reduce the gap between material-level performance and usable system output. However, these concepts should remain classified as exploratory until their advantages are demonstrated through complete module fabrication, prolonged high-temperature exposure, and application-relevant testing.

**Figure 5:**
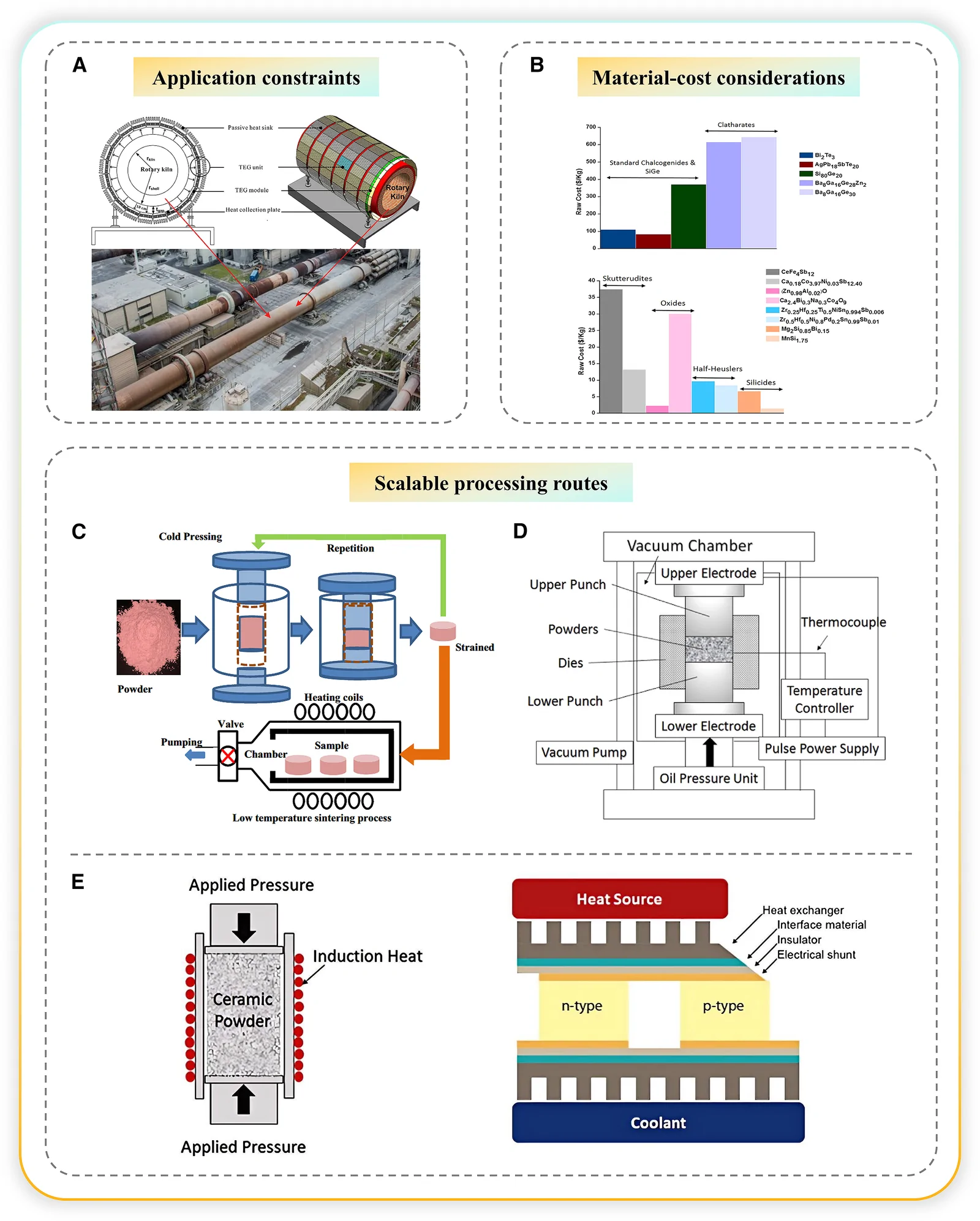
Application constraints, material and manufacturing cost factors, and representative processing routes for high-temperature thermoelectric generators (A) Waste heat recovery from rotary kiln surface using TEG.^235^ Copyright 2024, Elsevier. (B) Cost of various thermoelectric (TE) materials based on the raw material costs of the constituent elements.^271^ Copyright 2016, Elsevier. (C) A schematic illustration of cold-pressing with low-temperature sintering process of TE materials.^271^ Copyright 2016, Elsevier. (D) Schematic illustration of SPS technique for sintering TE materials.^271^ Copyright 2016, Elsevier. (E) Schematic illustration of hot-pressing technique for TE materials.^271^ Copyright 2016, Elsevier.

The existing evidence also indicates different levels of technological maturity. Carrier optimization, band engineering, diffusion barriers, and dense bulk processing have already demonstrated effectiveness at both material and module levels. Nanostructuring is effective for reducing lattice thermal conductivity, but its high-temperature stability and manufacturing reproducibility must be verified. Emerging concepts such as topological thermoelectrics, self-healing interfaces, and data-driven material discovery are strategically promising, although module-level and long-term evidence remains limited. Future studies should therefore distinguish material-level performance records from technologies that have been validated under realistic module and application conditions. Figure 5 links representative application constraints with material cost and manufacturing routes, emphasizing that scalable high-temperature thermoelectric generation requires coordinated progress in material preparation, module fabrication, and heat-source integration. It summarizes the engineering pathway from application constraints to scalable manufacturing. Figure 5A illustrates heat-source integration constraints using rotary-kiln waste-heat recovery as an example. Figure 5B compares the relative raw-material costs of representative thermoelectric systems, whereas Figures 5C–5E present representative densification routes. Together, these panels show that the commercialization of high-temperature TEGs depends on matching application conditions, material cost, and manufacturing route rather than optimizing material ZT alone.

## Conclusion

This review summarizes the development of thermoelectric materials for low-, medium-, and high-temperature power-generation applications and examines representative device and module studies that demonstrate the translation of material properties into practical electrical output. Across the three temperature regimes, the dominant design constraints shift progressively from limited temperature differences and thermal coupling to broad-range transport performance, interface stability, and high-temperature environmental reliability.

Low-temperature research is dominated by Bi_2_Te_3_-based compounds, Ag_2_Se, and organic or hybrid materials. In this regime, practical performance depends not only on material power factor but also on flexibility, thermal contact, parasitic heat loss, and electrical contact resistance. Medium-temperature materials, including PbTe-, GeTe-, and Mg_3_Sb_2_-based systems, have achieved high peak and average zT values through carrier optimization, band engineering, and multiscale phonon scattering. Their application in generators additionally requires compatible p-type and n-type legs, stable diffusion barriers, low-resistance contacts, and reliable segmented-module architectures.

At high temperatures, HH alloys, skutterudites, SiGe alloys, Cu_2_X compounds, Zintl phases, and thermoelectric oxides offer complementary advantages. However, peak material ZT alone is insufficient to predict generator performance. Oxidation, elemental volatilization, interfacial reactions, thermal-expansion mismatch, joining, packaging, and thermal management can determine module efficiency and operational lifetime. Material performance should, therefore, be evaluated together with average ZT, atmosphere-dependent stability, contact resistance, thermal-cycling durability, and module-level conversion efficiency.

Future progress will require coordinated optimization of materials, interfaces, modules, and heat-source integration. Near-term priorities include standardized long-term stability testing, chemically stable low-resistance contacts, reliable high-temperature packaging, and scalable fabrication. Data-driven material discovery, near-net-shape manufacturing, adaptive interfaces, and application-specific thermal-management structures may further accelerate development. Within this material-centered framework, the transition from laboratory performance records to reliable generator systems will depend on demonstrating long-term operation under realistic thermal, mechanical, and environmental conditions.

## Limitations of the study

The temperature boundaries adopted in this review provide a practical framework for comparing thermoelectric materials and generators but are not absolute, because the operating ranges of several material families overlap. The review is primarily material centered and, therefore, does not provide an exhaustive treatment of heat-exchanger design, power electronics, or industrial control. In addition, quantitative comparisons among published generators are limited by differences in module geometry, temperature boundary conditions, contact resistance, efficiency definitions, aging protocols, and reporting standards. Long-duration module-level data remain particularly limited for emerging high-temperature materials, restricting direct assessment of their technological maturity.

## Acknowledgments

This work is supported by Fundamental and Interdisciplinary Disciplines Breakthrough Plan of the 10.13039/501100002338Ministry of Education of China (JYB2025XDXM210), the 10.13039/501100001809National Natural Science Foundation of China (no. 52505633), 2025 Shaanxi Province Talent Support Program (2025SQBC001), and the Fundamental Research Funds for the Central Universities (xzy012025005).

## Author contributions

Conceptualization, Z.L., B.T., and Z.J.; investigation and literature curation, Z.L., J.G., J.J., C.W., K.W., Q.H., Q.W., and Z.Z.; formal analysis and synthesis, Z.L., J.G., and B.T.; visualization, Z.L., J.J., and J.G.; writing – original draft, J.G.; writing – review and editing, Z.L., J.G., J.J., C.W., K.W., Q.H., Q.W., Z.Z., B.T., and Z.J.; supervision, B.T. and Z.J.; project administration, B.T.; funding acquisition, B.T. and Z.J. All authors reviewed and approved the final version of the manuscript.

## Declaration of interests

The authors declare no competing interests.
